# Effects of ligand tuning and core doping of atomically precise copper nanoclusters on CO_2_ electroreduction selectivity

**DOI:** 10.1038/s42004-022-00779-0

**Published:** 2022-12-19

**Authors:** Mei Ding, Li Tang, Xiaoshuang Ma, Caixia Song, Shuxin Wang

**Affiliations:** College of Materials Science and Engineering, Qingdao University of Science and Technology, Qingdao, 266042 P. R. China

**Keywords:** Inorganic chemistry, Electrocatalysis, Nanoparticles

## Abstract

Atomically precise nanoclusters (NCs) provide opportunities for correlating the structure and electrocatalytic properties at atomic level. Herein, we report the single-atom doping effect and ligand effect on CO_2_ electroreduction (eCO_2_RR) by comparing monogold-doped Au_1_Cu_24_ and homocopper Cu_25_ NCs protected by triphenylphosphine or/and tris(4-fluorophenyl)phosphine. Catalytic results revealed that the electronic distribution of Cu_25_ NCs is enormously contracted by doping Au atoms, entitling it to exhibit the unique inhibition of hydrogen evolution reaction. And the inductive effect of ligand strongly favors the formation of formate in eCO_2_RR. Overall, this work will provide guidance for the rational design of the copper-based catalysts in the eCO_2_RR.

## Abstract (web summary)

Tuning the metal core and ligand environment of atomically precise nanoclusters enables the correlation of structural and electrocatalytic properties at an atomic level. Here, single-atom doping and ligand tuning of atomically precise copper clusters is shown to be an effective route to tuning CO_2_ electroreduction activity and selectivity.

## Introduction

Electrochemical CO_2_ reduction reaction (eCO_2_RR) has been attracting intensive research efforts, as it can convert CO_2_ to value-added chemicals in a carbon neutral route^1–4^. Due to the chemical inertness of CO_2_, sufficient activation energy is necessarily needed for breaking C–O bond and producing new carbon chemicals. Therefore, it is imperative for us to develop efficient catalysts to lower activation energy. So far, several transition metal-based catalysts have been successfully synthesized with high activity, but the specific selectivity for reduction products is relatively poor^5,6^.

Ligand-protected atomically precise metal nanoclusters (NCs) have been widely used in the field of catalysis^7–12^, owing to their unique properties and well-defined structures^13^. Recently, the catalysis application has expanded to eCO_2_RR to explore the formation mechanism of the reduction products, involving morphology^14^, active center^15–20^, size effect^21^, alloy effect^22–25^, electronic structure^26,27^, ligand type^28–31^, and so on. To be specific, Wang and co-workers reported that [Au_22_H_3_(dppe)_3_(PPh_3_)_8_]^3+^ exhibited higher selectivity towards CO (*FE* = 92.7% at −0.6 V) than that of [Au_11_(dppe)_5_]^3+^ (*FE* = 70.6%), in which the former was consisted of two Au_11_ units, indicating the structure of NCs plays an important role in electrochemical performance^32^. For another case, Pt_1_Au_37_(SCH_2_Ph^*t*^Bu)_24_ (15 electrons) was fabricated by doping mono-Pt into the kernels of Au_38_(SCH_2_Ph^*t*^Bu)_24_ (14 electrons), leading to a broader HOMO-LUMO gap and increased selectivity of CO, while two Pt atoms doped Pt_2_Au_37_(SCH_2_Ph^*t*^Bu)_24_ (16 electrons) was less active than Pt_1_Au_37_ for eCO_2_RR^26^. Furthermore, fine tuning of the NCs surface by ligand modification could enhance the selectivity for eCO_2_RR. For instance, Jin’s group found that the thiolated Au_25_ NCs showed an excellent selectivity of CO_2_ to CO, while the selenolated Au_25_ with the same metal packing inclined to the HER process^31^. Recently, Zang et al. reported that, Au_28_(C_2_B_10_H_11_S)_12_(C_4_H_8_S)_4_Cl_4_ exhibited higher *FE* of CO (98.5%) than that of the alkynyl protected Au_28_ due to the stripping of surface ligand to expose more active sites^28^.

Cu-based catalysts are considered to be the most promising eCO_2_RR catalysts because of their suitable adsorption energy for CO_2_ with H^33,34^. However, to our best knowledge, only a few cases about Cu NCs being used in eCO_2_RR have been reported. For example, Jiang and co-works used Cu_32_H_20_{S_2_P(O^*i*^Pr)_2_}_12_ as catalyst showing high selectivity of CO_2_ to HCOOH (*FE* = ~90%), further verified that the HCOOH formation proceeds via the lattice-hydride mechanism through density functional theory calculations and experiments^35^. Subsequently, Zang’s group found that the ditetrahedron-shaped Cu_8_ NCs exhibited ultra-high selectivity of HCOOH (*FE* = ~92%), which was twice as high as that of the cube-shaped Cu_8_ NCs isomers^36^. Recently, our group reported that, the formation of surface hydride played a significant role in triggering the formation and stabilization of HCOO* on the Ag-Cu active center, leading to the exclusive formation of formate in the Cu-containing NCs^37^. However, hydrogen evolution reaction (HER), as the competing reaction of eCO_2_RR, is inevitable in electroreduction with Cu-based NCs. Therefore, will the electronic distribution of NCs impact the composition of reduction products? How to effectively inhibit HER meanwhile promote eCO_2_RR? What is the relationship between the selectivity of C_1_ products (CO vs. HCOOH) and NCs structure? These questions form the aim and motivation of the current investigation.

Herein, we investigated the effects of the metal kernel and ligand on eCO_2_RR at the atomic level by employing four different [M@Cu_24_H_22_(PR_3_)_12_]^+^ (M = Au or Cu; R = -PPh_3_ or -*p-*FPPh_3_) NCs covered by two kinds of phosphine ligands, including [Cu_25_H_22_(Ph_3_P)_12_]^+^ (Cu_25_-(Ph)_3_P hereafter), [Cu_25_H_22_(*p-*FPh_3_P)_12_]^+^ (Cu_25_-(*p-*FPh)_3_P hereafter), [AuCu_24_H_22_(Ph_3_P)_12_]^+^ (AuCu_24_-(Ph)_3_P hereafter) and [AuCu_24_H_22_(*p-*FPh_3_P)_12_]^+^ (AuCu_24_-(*p-*FPh)_3_P hereafter). The Au-doped M@Cu_24_ NCs show preference towards the products of the eCO_2_RR, whereas homogeneous parent copper NCs tend to HER, indicating the metal dopants play a significant role in the electrochemical reactions. The kind of ligands also influences the selectivity of product in eCO_2_RR. As a comparison, the NCs catalysts protected by fluoro-substituted phosphonate ligands gave rise to a more enhanced activity for the electrochemical process of CO_2_-to-formate conversion at more positive potential. This work will provide guidance for the rational design of the copper-based catalysts for the eCO_2_RR.

## Results and discussion

### Synthesis and characterization of [M@Cu_24_H_22_(PR_3_)_12_]^+^ (M = Au or Cu; R = -PPh_3_ or -*p-*FPPh_3_) NCs

The four titled M@Cu_24_ NCs were synthesized through our previously reported methods with minor modifications^38^. The [Cu_25_H_22_(PPh_3_)_12_]Cl was firstly emerged as the by-product of Cu_18_ in C_6_H_6_ solvent reported by Hayton group^39^. In this work, compared with our previously reported method, we have improved the selectivity of these four target M@Cu_24_ NCs through the synthetic modification, which was a high-temperature reduction method using sodium borohydride under inert atmosphere. Note that the minor modifications, which were critical for the formation of these four M@Cu_24_ NCs with high yield. And we recorded the time-resolved UV–vis absorbance spectra of the four NCs to monitor the formation process, respectively. Note that, the absorption feature of Cu_25_-(Ph)_3_P and AuCu_24_-(Ph)_3_P are appeared at 1 h from the beginning (Supplementary Fig. 1a and 1b). Moreover, the four NCs could be controlled synthetized apace at 40 ^o^C with high yields. For instance, the fingerprint absorbance peaks of tris(4-fluorophenyl) phosphine-protected AuCu_24_-(*p-*FPh)_3_P (423 nm and 535 nm) and Cu_25_-(*p-*FPh)_3_P (454 and 619 nm) are observed within 1 h of the reaction (Supplementary Fig. 1c and 1d). Polyhydrido copper clusters with Cu(0) character obtained by high-temperature reduction, which may be due to the Cu–H bond heterolysis and Cu atoms nucleation promoted by the fast Pedesis.

**Fig. 1:**
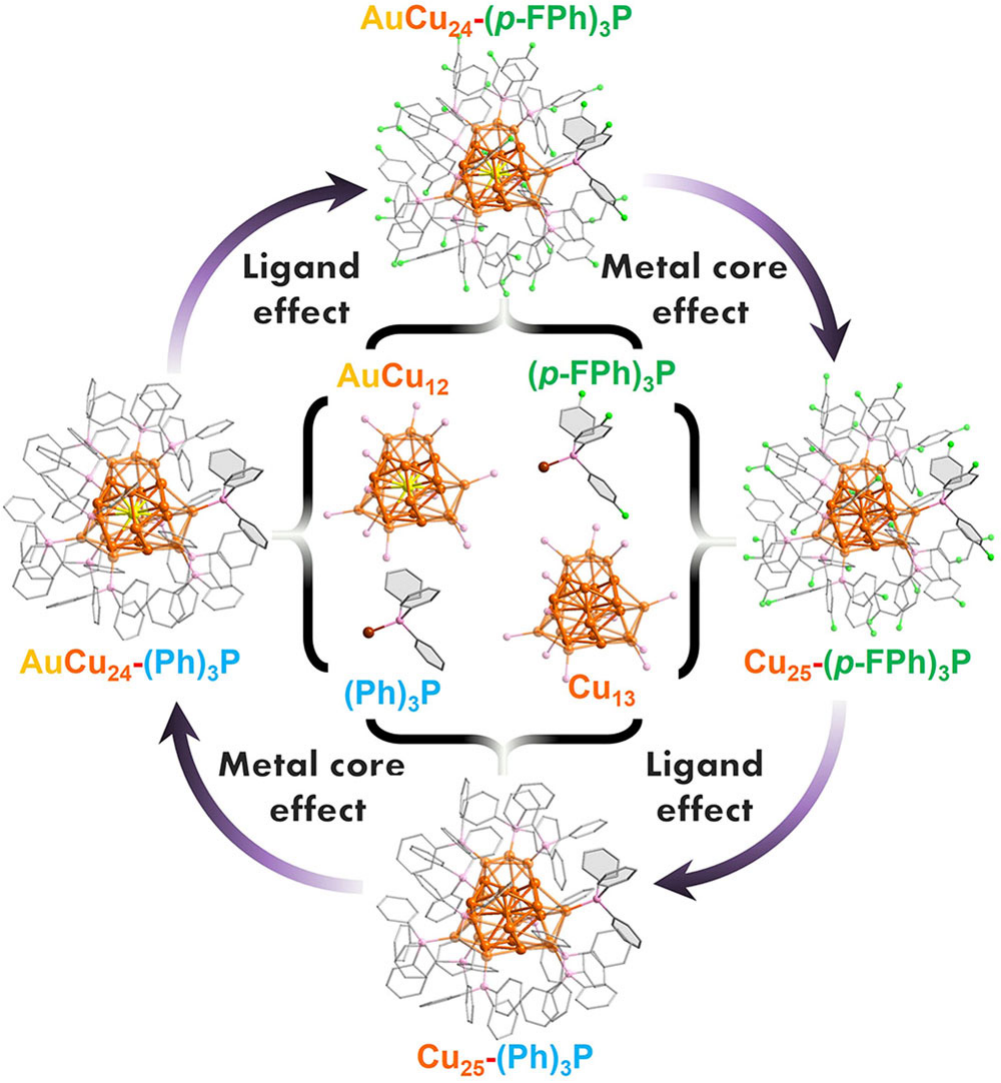
Total structures and anatomy of Cu_25_-(Ph)_3_P, Cu_25_-(*p*-FPh)_3_P, AuCu_24_-(Ph)_3_P and AuCu_24_-(*p*-FPh)_3_P. Color code: orange, Cu; yellow, Au; green, F; violet, P; gray, C. The H atoms are omitted for clarity.

In our previous work, the crystal structure and optical properties of the four NCs have been compared^38^. According to these reported crystallographic information and X-ray crystallography analysis of four clusters^38,39^, the four NCs have quite similar M@Cu_12_@Cu_12_ metal framework and surface motif arrangements (Fig. 1), providing an ideal platform to probe the electronic structure effect of the catalysts on the eCO_2_RR. All the four NCs share the same 13-atom M@Cu_12_ (M = Cu or Au) icosahedron kernel, which is covered by four Cu_3_(R_3_P)_3_ units (i.e., Cu_3_(Ph_3_P)_3_ or Cu_3_(*p-*FPh_3_P)_3_). The detailed synthetic procedure can be found in the “Methods” section, and the relevant characterization elucidation will be discussed next.

The electron distribution and composition of the four NCs can be revealed by UV–vis spectra and X-ray photoelectron spectroscopy (XPS). As displayed in Supplementary Fig. 2a and 2c, Cu_25_-(Ph)_3_P shows two distinct absorption peaks located at around 459 and 635 nm, while Cu_25_-(*p-*FPh)_3_P presents a little bit blue-shift (454 and 619 nm), due to electron-withdrawing ligands leading to an increased band gap. Similar absorption peak blue-shift also occurs in AuCu_24_-(Ph)_3_P (437 and 580 nm) and AuCu_25_-(*p-*FPh)_3_P (423 and 535 nm) (Supplementary Fig. 2b and 2d). Moreover, the two mono-Au-doping alloy NCs are brown in dichloromethane, in contrast with both Cu_25_-(Ph)_3_P and Cu_25_-(*p-*FPh)_3_P presenting a light green visible to the naked eye. The electronic structures of these four NCs were subsequently probed by X-ray photoelectron spectroscopy (XPS), and the results are presented in Supplementary Figs. 3, 4 and Supplementary Table 1. As depicted in Supplementary Figs. 3a, 3c, 4a and 4c, the XPS survey scan profiles confirmed the co-existence of the Cu, P, and C elements in Cu_25_-(Ph)_3_P; the Au, Cu, P, and C elements in AuCu_24_-(Ph)_3_P; Cu, P, F and C elements in Cu_25_-(*p-*FPh)_3_P; Au, Cu, P, F and C elements in AuCu_24_-(*p-*FPh)_3_P, respectively. The Au/Cu atomic ratio of AuCu_24_-(Ph)_3_P and AuCu_24_-(*p-*FPh)_3_P were estimated as 1.03/24.15 and 1.16/24.21, in good agreement with the theoretical value (1/24). As illustrated in Supplementary Figs. 3b, 3d, 4b and 4d, the binding energies of Cu 2p_3/2_ peaks in AuCu_24_-(Ph)_3_P (932.56 eV) and AuCu_24_-(*p-*FPh)_3_P (932.62 eV) are much higher than that of Cu_25_-(Ph)_3_P (932.42 eV) and Cu_25_-(*p-*FPh)_3_P (932.48 eV), suggesting that one gold atom doped can significantly affect the electron distribution of the parent homogenous Cu_25_ NCs. Compared with homogenous Cu_25_ NCs, the free valence electrons of the AuCu_12_ core enormously shrunk inward by one gold doping, leading to a fact that Cu on the surface were electropositive. Furthermore, the binding energy of relatively electron-withdrawing ligands covered AuCu_24_-(*p-*FPh)_3_P (932.62 eV) was slightly higher than that of AuCu_24_-(Ph)_3_P (932.56 eV), and it means that more electropositive Cu atoms could be observed in relatively electron-withdrawing ligands coved M@Cu_24_ NCs. Meanwhile, the binding energies of the Au 4f_7/2_ peaks in AuCu_24_-(*p-*FPh)_3_P (84.14 eV) and AuCu_24_-(*p-*FPh)_3_P (84.48 eV) are on a higher-energy side (oxidation side) relative to that of Au(0) (84.0 eV), demonstrating that the incorporated Au is partially oxidized (Supplementary Figs. 3e and 4e).

### Electrocatalytic CO_2_ reduction performance

Electrochemical CO_2_ reduction reaction (shorted for eCO_2_RR) was envisioned to measure the catalytic performances of these title NCs. Given the abundant surface defects and functional groups of acid multi-walled carbon nanotubes (CNTs), the four NCs were deposited onto CNTs with 50% wt. loading to form the AuCu_24_-(Ph)_3_P/CNTs, Cu_25_-(Ph)_3_P/CNTs, AuCu_24_-(*p-*FPh)_3_P/CNTs and Cu_25_-(*p-*FPh)_3_P/CNTs electrocatalysts. Noted that, no nanoparticles were observed on the surface of four electrocatalysts, which corresponds to the X-ray diffraction (XRD) pattern (Supplementary Fig. 5). It was found that CO and H_2_ were the only two gaseous products under all applied potential (without IR correction) versus the reversible hydrogen electrode (RHE) by gas chromatography (GC). In addition, ^1^H-NMR spectra verified liquid products (Supplementary Fig. 6). The linear scanning voltammetry (LSV) were first conducted for the four catalysts. As depicted in Supplementary Fig. 7, both AuCu_24_-(Ph)_3_P/CNTs and AuCu_24_-(*p-*FPh)_3_P/CNTs exhibit a much higher current density and a more positive onset potential in CO_2_ saturated aqueous 0.5 M KHCO_3_ solution than in the one of N_2_ purged, indicating that the two mono-Au-doped clusters have much higher CO_2_ reduction activity. Cu_25_-(Ph)_3_P/CNTs and Cu_25_-(*p-*FPh)_3_P/CNTs, almost the same current density are observed at all of the test potentials, implying that HER rather than eCO_2_RR is the major reduction process for both Cu_25_ NCs electrocatalysts.

**Fig. 2:**
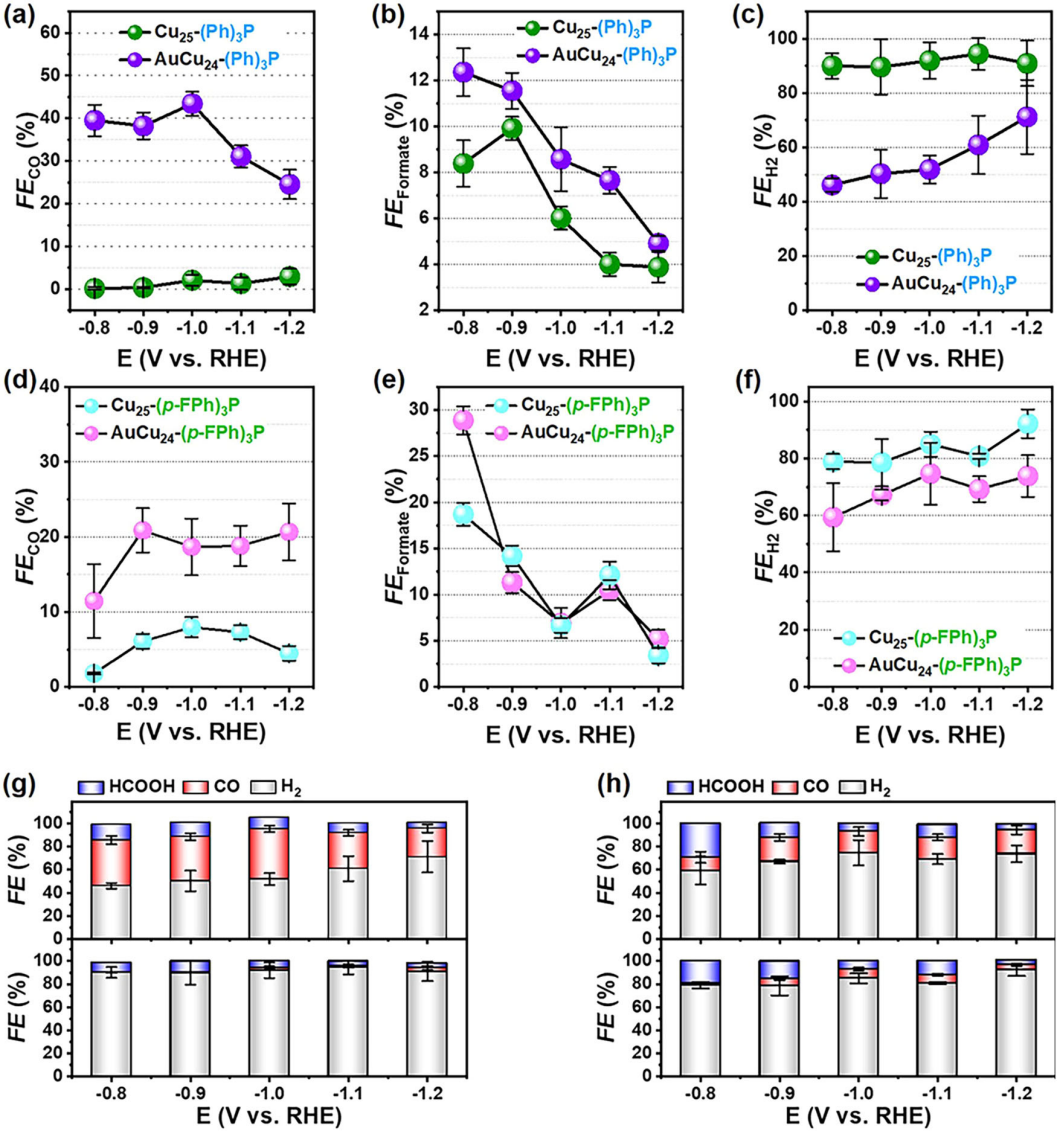
The electrocatalytic performance of the four M@Cu_24_ (M = Au/Cu) NCs in eCO_2_RR. **a**, **d** CO, **b**, **e** formate and **c**, **f** H_2_ faradaic efficiency of AuCu_24_-(Ph)_3_P, Cu_25_-(Ph)_3_P, and Cu_25_-(*p-*FPh)_3_P, AuCu_24_-(*p-*FPh)_3_P, respectively. FEs for various eCO_2_RR products obtained on **g** AuCu_24_-(Ph)_3_P, Cu_25_-(Ph)_3_P, and **h** AuCu_24_-(*p-*FPh)_3_P, Cu_25_-(*p-*FPh)_3_P. The error bars represent the standard deviation of three tests at the same test potential.

For these four electrocatalysts, one-metal-atom and ligand difference can cause remarkable eCO_2_RR catalytic performance discrepancy, and a strong metal core effect (Cu_13_ vs. AuCu_12_) and ligand effect (Ph_3_P vs. FPh_3_P) is observed. As illustrated in Fig. 2a–c, AuCu_24_-(Ph)_3_P/CNTs exhibits the highest CO faradaic efficiency (FE_CO_) of 45.6% at −1.0 V, while H_2_ is the main product (FE_H2_ >80%) of the Cu_25_-(Ph)_3_P/CNTs in all tested potential ranges and the FE of CO and formate is only ~0–3.7% and ~6.1–10.1% at −0.8 to −1.0 V. It was due to the core effect (Au vs. Cu), suggesting that the mono-gold doped AuCu_24_ can selectively reduce CO_2_ to high value-added carbon products. In contrast, the homogeneous Cu-based NCs, because of the high Cu-H binding energy, could be the active center for HER. Similarly, AuCu_24_-(*p-*FPh)_3_P/CNTs and Cu_25_-(*p-*FPh)_3_P/CNTs, which had only one-metal-atom difference from each other, displayed FE_CO_ content only 11.8% to 20.6% and 2.1% to 8.2% in the entire tested potential, respectively (Fig. 2d). However, compared with triphenylphosphine covered NCs, AuCu_24_-(*p-*FPh)_3_P/CNTs (FE_formate _= 30.6%) and Cu_25_-(*p-*FPh)_3_P/CNTs (FE_formate _= 20.3%) have higher FE of formate, which were several times more than that of AuCu_24_-(Ph)_3_P and Cu_25_-(Ph)_3_P (Fig. 2e). Futhermore, the selectivity btween C_1_ and H_2_ product of Cu_25_-(*p-*FPh)_3_P/CNTs (FE_H2 _= 80.2 to 90.4%) and AuCu_24_-(*p-*FPh)_3_P/CNTs (FE_H2 _= 59.3–70.6%) are similiar with triphenylphosphine counterparts (Fig. 2f). As shown in the Fig. 2g, the FE_CO+formate_ on AuCu_24_-(Ph)_3_P/CNTs of 55.9% is almost four times more than that of Cu_25_-(Ph)_3_P/CNTs (FE_CO+formate _= 14.8%) at the tested potential of −0.8 V, while the Cu_25_-(*p-*FPh)_3_P/CNTs had lower FE_CO+formate_ (20.8%), which were about a half that of AuCu_24_-(*p-*FPh)_3_P/CNTs (FE_CO+formate _= 40.5%) (Fig. 2h). Noted that, the formate selectivity on the two tris(4-fluorophenyl)phosphine protected NCs were two times more than that of AuCu_24_-(Ph)_3_P/CNTs and Cu_25_-(Ph)_3_P/CNTs, indicating that the ligand effect had a momentous effect on selectively reducing CO_2_ to C_1_ products.

Meanwhile, the CO partial current density (j_CO_) increased with the increasement of applied potential for the four electrocatalysts (Supplementary Fig. 8a and 8d). Both of mono-gold-doped NCs had a much larger j_CO_ value than those of Cu_25_-(Ph)_3_P/CNTs and Cu_25_-(*p-*FPh)_3_P/CNTs at all potentials, further manifesting unique advantage of Au-doped NCs for converting CO_2_ into CO exclusively. Furthermore, the partial current density of formate (j_formate_) for AuCu_24_-(Ph)_3_P/CNTs and Cu_25_-(Ph)_3_P/CNTs increased with the increasement of tested potential (Supplementary Fig. 8b). However, the j_formate_ of the two tris(4-fluorophenyl)phosphine covered NCs exhibited the same trend with each other, that is, it first increased then decreased, and the maximal value is 3.2 mA cm^−2^ for Cu_25_-(*p-*FPh)_3_P/CNTs and 2.6 mA cm^−2^ for AuCu_24_-(*p-*FPh)_3_P/CNTs at −1.1 V, which is much higher than that of triphenylphosphine counterparts (Supplementary Fig. 8e). This is mainly attributed to that the HER process became dominant at more negative potentials. Note that, both of Au-doped NCs had smaller partial current density of H_2_ (j_H2_) than that of corresponding Cu_25_ NCs, indicating that one gold atom doping could increase the selectivity for deducing CO_2_ to C_1_ product (Supplementary Fig. 8c and 8f).

**Fig. 3:**
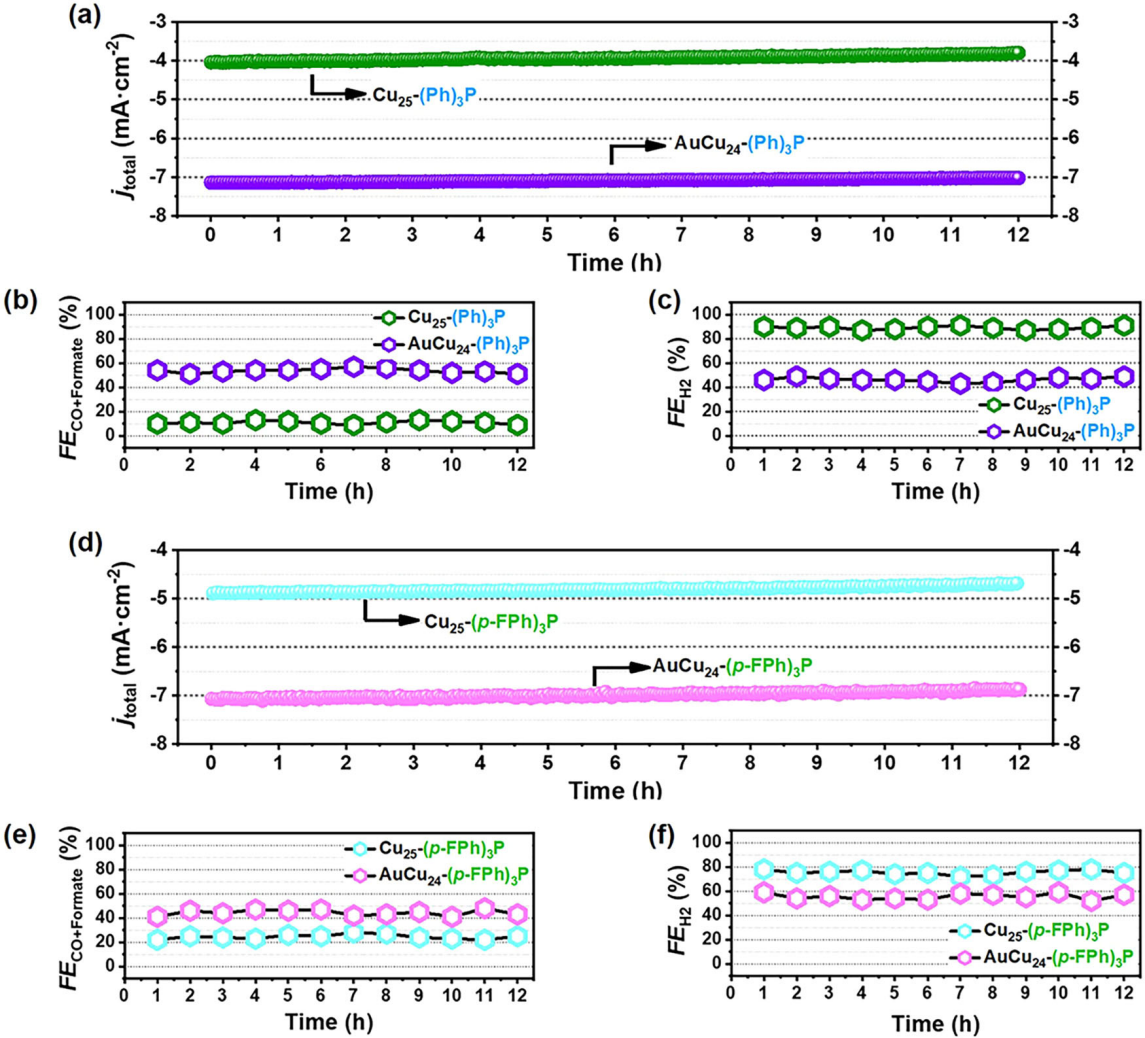
Long-term stability of Cu_25_-(Ph)_3_P, AuCu_24_-(Ph)_3_P, Cu_25_-(*p-*FPh)_3_P and AuCu_24_-(*p-*FPh)_3_P at −0.80 V. **a**, **d** i-t curve; FEs of **b**, **e** C_1_ products and **c**, **f** H_2_ at different time.

Stability is a significant index for evaluating well-defined NCs electrocatalysts in eCO_2_RR, hence the long-term stability of the four catalysts were tested at −0.8 V, respectively. As illustrated in Fig. 3a, d, the current density and corresponding FE value of two mono-Au doped NCs remained almost unchanged (j_total _= −7.13 to −6.91 mA cm^−2^ for AuCu_24_-(Ph)_3_P/CNTs; j_total_ from −7.05 to −6.90 mA cm^−2^ for AuCu_24_-(*p-*FPh)_3_P/CNTs) after 12 hours of continuous operation, indicating robust long-term durability. However, under the same conditions, the current density of Cu_25_-(Ph)_3_P/CNTs and Cu_25_-(*p-*FPh)_3_P/CNTs decreased about 5.7% (from −4.04 to −3.81 mA cm^−2^) and 4.3% (from −4.90 to −4.69 mA cm^−2^), respectively. Meanwhile, the FE_CO+formate_ and FE_H2_ of the four electrocatalysts are almost unchanged during the whole time (Fig. 3b, c, e, f). Furthermore, the morphologies of these NCs after eCO_2_RR are almost the same as before (Supplementary Fig. 9). These results indicate that, the majority of NCs electrocatalysts can be well preserved during the eCO_2_RR process. Using the fingerprint absorbance peak (635 nm for Cu_25_-(Ph)_3_P/CNTs; 580 nm for AuCu_24_-(Ph)_3_P/CNTs; 619 nm for Cu_25_-(*p-*FPh)_3_P/CNTs; 535 nm for AuCu_24_-(*p-*FPh)_3_P/CNTs) as the metric, the absorbance change can be quantified and employed to estimate the recovery rate (Supplementary Fig. 10). Notably, the entire absorbance feature of the four NCs kept constantly, though the intensity of the characteristic peaks was decreased at varying degrees under different potentials. At the −0.8 V, all the recovery rate of four catalysts were over 50%, which was in good agreement with the finding in the *i*-t test and the calculated results as summarized in Supplementary Table 2.

The electrochemically active surface area (ECSA) was measured to further reveal the reason for the difference in the catalytic performance of the four clusters in eCO_2_RR. The current density of Cu_25_-(Ph)_3_P, AuCu_24_-(Ph)_3_P, Cu_25_-(*p-*FPh)_3_P, and AuCu_24_-(*p-*FPh)_3_P at different scan rate ranging from 20 to 100 mV s^−1^ were recorded and shown in Supplementary Fig. 11, and based on that, the double-layer capacitance (C_dl_) of the four NCs were evaluated to be 1.27, 1.21, 0.99 and 0.94 mF, respectively. Thus, the ECSA of Cu_25_-(Ph)_3_P, AuCu_24_-(Ph)_3_P, Cu_25_-(*p-*FPh)_3_P, and AuCu_24_-(*p-*FPh)_3_P were calculated to be 31.75, 30.25, 24.75 and 23.50 cm^2^, respectively. Therefore, the number of active sites of the three clusters can be arranged in descending order as Cu_25_-(Ph)_3_P > AuCu_24_-(Ph)_3_P > Cu_25_-(*p-*FPh)_3_P > AuCu_24_-(*p-*FPh)_3_P, which was attributed to the number of active sites scales with the ligand removal. Furthermore, the electrochemical impedance (EIS) was carried out to explore the charge transport properties of the four NCs at the electrode/electrolyte interface (Supplementary Fig. 12). The Nyquist plot of both homogeneous Cu_25_ NCs exhibited a much smaller the semicircular diameters than that of mono-gold-doped AuCu_24_, and the former have a conductivity with lower interfacial charge-transfer resistance.

### Effect on electron-density distribution modulating of copper catalyst on the selectivity of C_1_ products (CO vs. formate) from eCO_2_RR

**Fig. 4:**
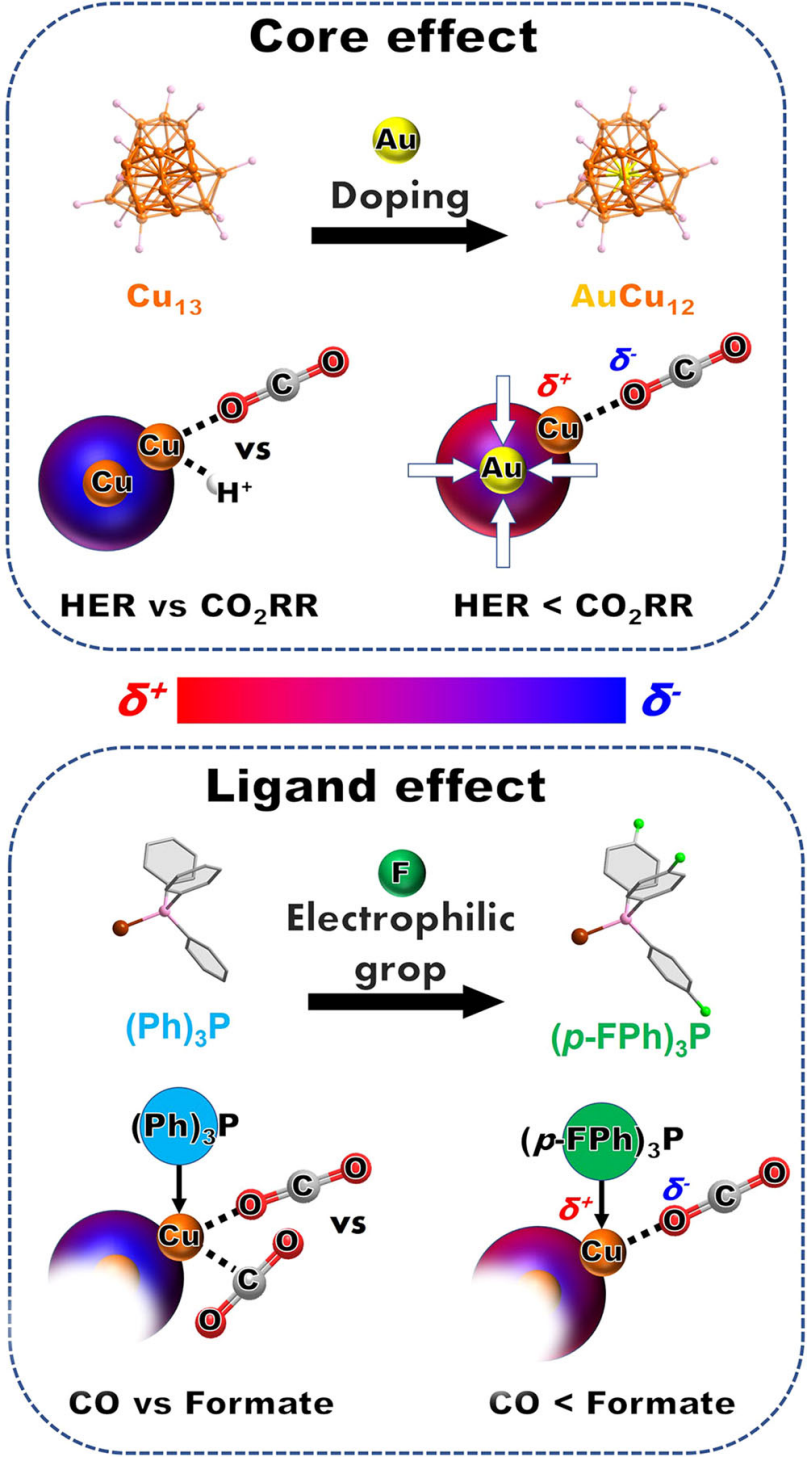
The scheme of electron distribution modulating on NCs by ligand tuning and core doping. **a** Core effect (Cu_13_ vs. AuCu_12_) and **b** ligand effect (triphenylphorsphine vs. tris(4-fluorophenyl)phosphine).

According to the catalytic performance above, we find that the metal core plays an important role in the two competitive reactions (HER vs. eCO_2_RR) in electroreduction selectivity, further, achieving the formation selectivity for C_1_ products (formate vs. CO) in eCO_2_RR by changing the ligand type. Get insight in the electron-density distribution of the M@Cu_24_ (Au/ Cu), as displayed in Fig. 4, core and ligand effect could be observed: (i) the Cu atom on the surface of mono-gold-doped AuCu_24_ have lower electron cloud density (δ^+^) and is more prone to eCO_2_RR, caused by the free valence electrons of the AuCu_12_ core to shrink inward; (ii) The more electropositive Cu atoms banding with electrophilic ligand are preferable to bind the O atom of CO_2_, and showing selectivity for formate. Experimentally, the XPS results reflected the positive charge distribution among the four NCs. By mono-gold doping, the exterior Cu atoms of AuCu_24_-(Ph)_3_P (932.56 eV) and AuCu_24_-(*p*-FPh)_3_P (932.62 eV) are much more electropositive than that of Cu_25_-(Ph)_3_P (932.42 eV) and Cu_25_-(*p*-FPh)_3_P (932.48 eV) (Supplementary Figs. 3b, 3d, 4b and 4d). It was noting that, HER was inevitable for the polyhydrido Cu NCs catalysts in electrochemical reduction, however, it was advisable to increase the selectivity toward to eCO_2_RR and reduce the H-source supply in the solution by electron-contracted element such as Au, Pt, Pd, etc. More importantly, using electrophilic ligand could not only raise the stability of NCs, and promote the selectivity of formate in eCO_2_RR.

## Conclusion

In summary, these four polyhydrido M@Cu_24_ (M = Au or Cu) NCs were controllably synthesized and used as electrocatalysts to probe the core and ligand effect for electroreduction selectivity. Significantly, the inert metal doping was propitious to eCO_2_RR, and the modification of the electrophilic ligand was efficient to enhance the selectivity for formate in eCO_2_RR. This work not only provides an efficient and simple strategy to prepare the polyhydrido Cu-based NCs, but also highlights the unique advantages of employing metal NCs as model catalysts to advance the fundamental mechanistic understanding toward electroreduction and beyond.

## Methods

### Chemicals and materials

The cupric (II) acetylacetonate (C_10_H_14_O_4_Cu), tetrachloroauric (III) acid (HAuCl_4_·3H_2_O, >99.99% metals basis), triphenylphosphine (Ph_3_P, ≥99%), tris(4-fluorophenyl)phosphine ((*p-*FPh)_3_P, ≥99%), NaBH_4_ (>98%) were received from Aldrich (Shanghai, China). Methanol, dichloromethane, and n-hexane were purchased from Aldrich (Shanghai, China). All reagents and solvents were commercially available and used without further purification. Pure water was purchased from Wahaha Co. Ltd. All glassware was thoroughly cleaned with aqua regia (HCl:HNO_3 _= 3:1, v-v), rinsed with copious pure water.

### Synthesis of [Cu_25_H_22_(Ph_3_P)_12_]^+^ nanoclusters (Cu_25_-(Ph)_3_P)

Firstly, Cu_25_-(Ph)_3_P NCs were prepared according to a reported protocol with some minor modifications^38^. Briefly, at 40 ^o^C under Ar atmosphere, a solution of cupric(II) acetylacetonate (60 mg) dissolved in a mixed solvent of methanol and dichloromethane in a volume ratio of 3:1. The color of the solution was ink blue. After 30 min, triphenylphosphine (100 mg) was added, and the solution changed slowly from ink blue to light blue. After that, 2 mL freshly aqueous NaBH_4_ (0.66 M in ice-cold water) was quickly added under vigorous stirring. The color of the solution slowly changed from light blue to yellow-green within 10 min. The reaction was aged for 2.5 h at room temperature. The solvent was then evaporated to give a yellow-green solid. After the reaction, the volume of the mixture was evaporated to dryness to give a black solid, which was successively washed with n-hexane and diethyl ether to remove the byproducts and excess triphenylphosphine, followed by extraction with dichloromethane and then dried by rotary evaporation. After diffusion of n-hexane to a dichloromethane solution at −4 ^o^C for about one week, black crystals were obtained (yield: ~40% based on copper salt).

### Synthesis of the [Cu_25_H_22_((*p-*FPh)_3_P)_12_]^+^ nanoclusters (Cu_25_-(*p-*FPh)_3_P)

The synthesis of Cu_25_-(*p-*FPh)_3_P was similar to that of Cu_25_-(Ph)_3_P and the synthetic procedures are identical until the addition of ligand, which triphenylphosphine was replaced with tris(4-fluorophenyl)phosphine. After that, 2 mL freshly aqueous NaBH_4_ (0.66 M in ice-cold water) was quickly added under vigorous stirring. The reaction was aged for 10 h at room temperature. After the reaction, the volume of the mixture was evaporated to dryness to give black solid, which was successively washed with n-hexane and diethyl ether to remove the byproducts and excess ligands, followed by extraction with dichloromethane and then dried by rotary evaporation. After diffusion of n-hexane to a dichloromethane solution at −4 ^o^C for about two days, black crystals were obtained (yield: ~70% based on copper salt).

### Synthesis of [AuCu_24_H_22_(Ph_3_P)_12_]^+^ nanoclusters (AuCu_24_-(Ph)_3_P)

Firstly, AuCu_24_-(Ph)_3_P was synthesized following the methods reported by us previously with some minor modifications^38^. Briefly, at 40 ^o^C under Ar, a solution of cupric(II) acetylacetonate (60 mg) and HAuCl_4_·3H_2_O (5 mg) dissolved in mixed solvent of methanol and dichloromethane in a volume ratio of 3:1. The color of the solution was ink blue. After 30 min, triphenylphosphine (100 mg) was added and the solution changed slowly from ink blue to light blue. After that, 2 mL freshly aqueous NaBH_4_ (0.66 M in ice-cold water) was quickly added under vigorous stirring. The color of the solution quickly turned reddish brown. The reaction was aged for 10 h at room temperature. The solvent was then evaporated to give a yellow-green solid. After the reaction, the volume of the mixture was evaporated to dryness to give black solid, which was successively washed with n-hexane and diethyl ether to remove the byproducts and excess triphenylphosphine, followed by extraction with dichloromethane and then dried by rotary evaporation. After diffusion of n-hexane to a dichloromethane solution at −4 ^o^C for about three days, black crystals were obtained (yield: ~30% based on copper salt).

### Synthesis of [AuCu_24_H_22_(*p-*FPh_3_P)_12_]^+^ nanoclusters (AuCu_24_-(*p-*FPh)_3_P)

The synthesis of AuCu_24_-(*p-*FPh)_3_P was similar to that of AuCu_24_-(Ph)_3_P and the synthetic procedures are identical until the addition of ligand, which triphenylphosphine was replaced with tris(4-fluorophenyl)phosphine. Then, 2 mL freshly aqueous NaBH_4_ (0.66 M in ice-cold water) was quickly added under vigorous stirring. The color of the solution quickly turned reddish brown. The reaction was aged for 10 h at 40 ^o^C. The solvent was then evaporated to give a reddish-brown solid. After the reaction, the volume of the mixture was evaporated to dryness to give a black solid, which was successively washed with n-hexane and diethyl ether to remove the byproducts and excess ligands, followed by extraction with dichloromethane and then dried by rotary evaporation. After diffusion of n-hexane to a dichloromethane solution at −4 ^o^C for about three days, black crystals were obtained (yield: ~80% based on copper salt).

### Electrochemical measurements

To prepare the catalyst sample, these four as-prepared M@Cu_24_ (M = Au/Cu) were loading on acidic multi-walled carbon (CNTs) with a mass ratio of 1 (5 mg NC and 5 mg CNTs). The catalyst ink was prepared by dispersing the sample in isopropyl alcohol under sonication for 5 min. Then 1 mL catalyst suspension and 10 μL Nafion (5 wt.%) were uniformly mixed as the final catalyst ink. Then, 40 μL catalytic ink was dropwise cast onto the carbon cloth (1.0 × 1.0 cm^2^) and dried at room temperature as the working electrode. All electrochemical measurements were carried out in a custom gas-tight H-cell with two compartments separated by Nafion 117 membrane. Each compartment contained 25 mL electrolyte (0.5 M KHCO_3_: pH =  7.2 when saturated with CO_2_, pH = 8.8 when saturated with N_2_) with ~10 mL headspace. The electrochemical measurements were carried out on an electrochemical workstation (CHI 760E) with Pt sheet as counter electrode and Ag/AgCl electrodes (KCl saturated) as reference electrode. All the potentials were calibrated to a reversible hydrogen electrode (RHE) according to the Nernst equation:

1 $$R({{{{{\rm{RHE}}}}}})={{{{{\rm{E}}}}}}({{{{{\rm{Ag}}}}}}/{{{{{\rm{AgCl}}}}}})+0.059\times {{{{{\rm{pH}}}}}}+0.197$$

The output of the gas flow from the cathode chamber was directed into a gas chromatograph instrument (GC3900Plus, RUI NENG) for identification and quantification of the gaseous products, which was purged for 30 min with an average rate of 10 mL min^−1^ (at room temperature and ambient pressure) prior to the test. The GC was installed with a thermal conductivity detector (TCD) to detect H_2_ and flame ionization detector (FID) to detect hydrocarbons. A methanizer (Agilent) was equipped in front of the FID for CO detection. High-purity Argon (99.9999%) was used as the carrier gas for all compartments of the GC.

The faradaic efficiency (*FE*_X_) and corresponding partial current density (*j*_X_) of X (X = CO or H_2_ or formate) were calculated as below:

2 $${{{{{{\rm{FE}}}}}}}_{X}=\frac{({N}_{i}\times {{{{{\rm{n}}}}}}\times {{{{{\rm{F}}}}}})}{{Q}_{t}}$$

3 $${j}_{X}=\frac{{{{{{{\rm{FE}}}}}}}_{X}\times {Q}_{t}}{{{{{{\rm{t}}}}}}\times {{{{{\rm{Area}}}}}}}$$

Where

*Q*_t _= total charge consumed in the electrochemical reaction

*N*_i _= the number of moles of the product (measured GC) *n* = the number of electrons transferred in the elementary reaction (*n* is 2 for CO, H_2_ and formate) *F* = the Faradaic constant (96485 C mol^−1^)

*t* = reaction time (s)

Area = geometry area of the electrode (1 cm^2^)

The electrochemically active surface area (ECSA) of the catalyst was obtained via the linear fit of the corresponding current density with respect to the scan rate (Fig. S10). The results show that double layer capacitance of electrode (C_dI_) for Cu_25_-(Ph)_3_P, AuCu_24_-(Ph)_3_P, Cu_25_-(*p*-FPh)_3_P and AuCu_24_-(*p*-FPh)_3_P are 1.27, 1.21, 0.99 and 0.94 mF, respectively. The ESCA can be calculated based on the following formula:

4 $${{{{{\rm{ECSA}}}}}}=\frac{{C}_{{{{{{\rm{dI}}}}}}}}{{C}_{s}}$$

where *C*_S_ is the specific capacitance of the sample or capacitance of an atomically smooth planar surface of the material per unit area under identical electrolyte conditions. And the average *C*_S_ value of 0.04 mF cm^−2^ for an ideal flat surface of the metal catalyst in alkaline solution reported by McCrory was chosen for the ECSA evaluation^40^.

For the morphology of four catalysts before and after eCO_2_RR, the NCs/CNTs loaded work electrodes were directly conducted by SEM measurement. For stability, the NCs/CNTs loaded carbon fibers were as work electrodes. The eCO_2_RR was allowed to proceed at −0.8 V (vs. RHE) for 30 min. After the test, the samples were rinsed with dichloromethane and collected for UV–vis characterization.

### General characterization

The surface chemical compositions and valence states were examined by X-ray photoelectron spectroscopy (XPS, Phi X-tool instrument). Scanning electronic microscopic (SEM) images were collected with a field-emission scanning electron microscope (FESEM, Merlin). UV–visible absorption spectra of clusters, dimer and tetramer were recorded on a Shimadzu 2600/2700 spectrophotometer.

## Supplementary information

Wang_PR File

Supplementary Information

## Data Availability

All data generated or analyzed during this study are included in this article (and its Supplementary Information files).
