# Supplementary material for: Effects of ligand tuning and core doping of atomically precise copper nanoclusters on CO2 electroreduction selectivity
Source: Commun Chem. 2022 Dec 19;5:172. doi: 10.1038/s42004-022-00779-0 (PMC9814636; doi:10.1038/s42004-022-00779-0)
Supplement: Supplementary file 2 — Supplementary Information [file 42004_2022_779_MOESM2_ESM.docx]

Supplementary Information for

Effects of ligand tuning and core doping of atomically precise copper nanoclusters on CO_2_ electroreduction selectivity

Mei Ding^1^, Li Tang^1^, Xiaoshuang Ma^1^*, Caixia Song^1^* & Shuxing Wang^1^*

^1^College of Materials Science and Engineering, Qingdao University of Science and Technology, Qingdao 266042 (P. R. China). Correspondence and requests for materials should be addressed to X.M. (email: [xiaoshuang_ma@qust.edu.cn](mailto:xiaoshuang_ma@qust.edu.cn)) or to C.S. (email: [songcaixia@qust.edu.cn](mailto:songcaixia@qust.edu.cn)) or to S.W. (email: [shuxin_wang@qust.edu.cn](mailto:shuxin_wang@qust.edu.cn))

**Table of Contents**

**Supplementary Figures**

**Supplementary Fig. 1:** Time resolved UV-vis absorbance spectra of four M@Cu_24_ (M= Au/ Cu) NCs.

**Supplementary Fig. 2:** Absorbance spectra of four purified M@Cu_24_ NCs.

**Supplementary Fig. 3:** XPS spectra of *A*uCu_24_-(Ph)_3_P and Cu_25_-(Ph)_3_P NCs.

**Supplementary Fig. 4:** XPS spectra of AuCu_24_-(*p*-FPh)_3_P and Cu_25_-(*p*-FPh)_3_P NCs.

**Supplementary Fig. 5:** XRD patterns of the four M@Cu_24_ NCs.

**Supplementary Fig. 6:** ^1^H-NMR spectra of the eCO_2_RR product in liquid phase.

**Supplementary Fig. 7:** LSV curves of four M@Cu_24_ NCs in an N_2_ and a CO_2_ saturated 0.5 M KHCO_3_ solution.

**Supplementary Fig. 8:** The corresponding H_2_, CO and formate partial current density for four M@Cu_24_ NCs.

**Supplementary Fig. 9:** SEM image of four M@Cu_24_ NCs before and after eCO_2_RR at -0.8 V.

**Supplementary Fig. 10:** UV-vis spectra of four M@Cu_24_ NCs before and after eCO_2_RR at -0.8 V.

**Supplementary Fig. 11:** CV and the corresponding linear curves of current density of four M@Cu_24_ NCs at a scan rate from 20 to100 mV·s^–1^.

**Supplementary Fig. 12:** EIS of four M@Cu_24_ NCs.

**Supplementary Tables**

**Supplementary Table 1:** The XPS data of Cu_25_-(Ph)_3_P, AuCu_24_-(Ph)_3_P, Cu_25_-(*p*-FPh)_3_P and AuCu_24_-(*p*-FPh)_3_P NCs.

**Supplementary Table 2:** Recovery of the four electrocatalysts after eCO_2_RR test at -0.80 V.

**Supplementary Figures**

**
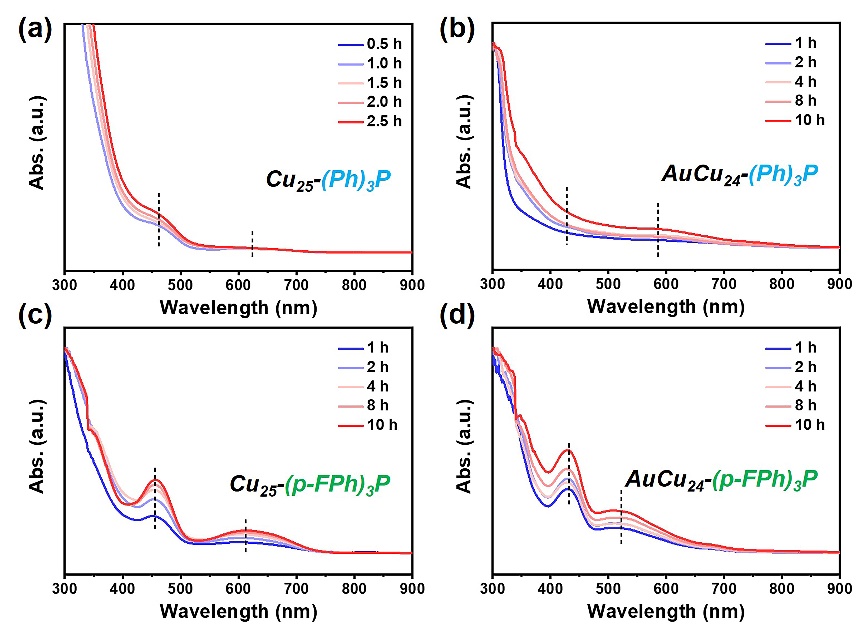
**

**Supplementary Fig. 1:** Time resolved UV-vis absorbance spectra of (a) Cu_25_-(Ph)_3_P, (b) AuCu_24_-(Ph)_3_P, (c) Cu_25_-(*p*-FPh)_3_P and (d) AuCu_24_-(*p*-FPh)_3_P.


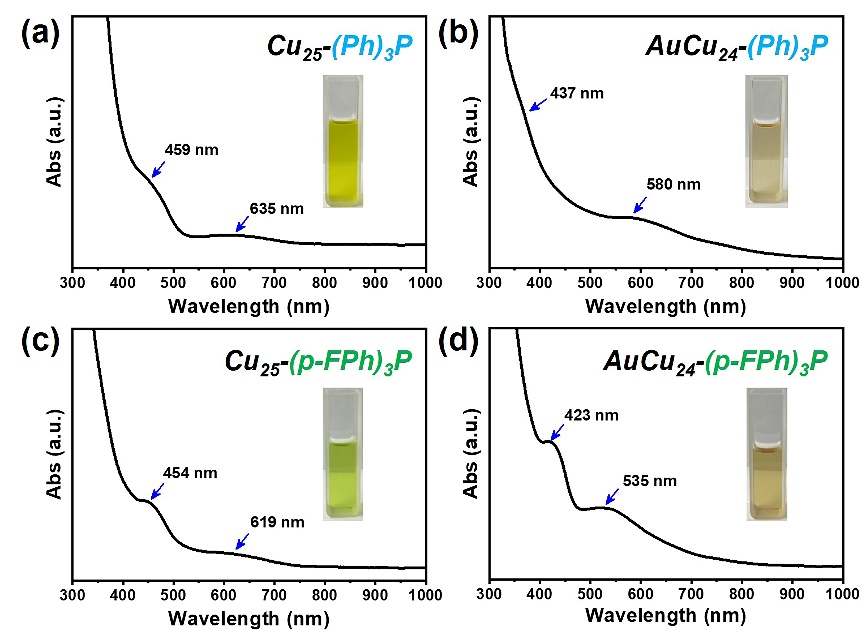


**Supplementary Fig. 2:** Absorbance spectra of (a) Cu_25_-(Ph)_3_P, (b) AuCu_24_-(Ph)_3_P, (c) Cu_25_-(*p*-FPh)_3_P and (d) AuCu_24_-(*p*-FPh)_3_P. Insets are the digital photographs of these four clusters in dichloromethane, respectively.

**
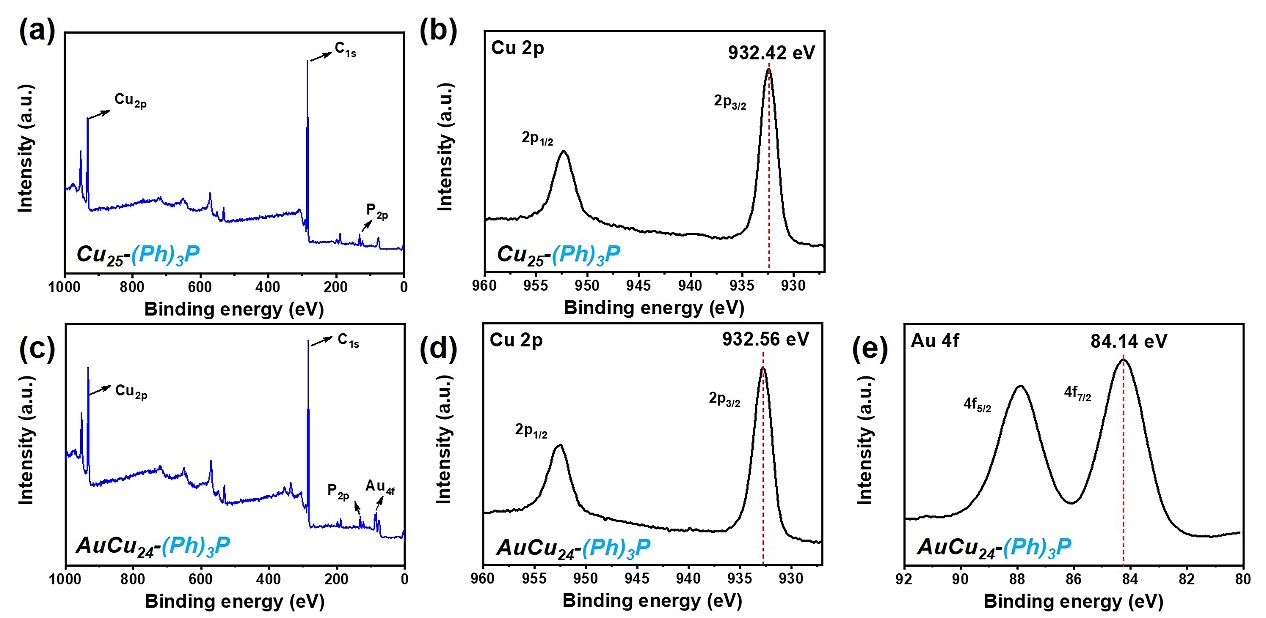
**

**Supplementary Fig. 3:** (a) The XPS survey scan spectra of (a) Cu_25_-(Ph)_3_P and (c) AuCu_24_-(Ph)_3_P. The core-level XPS spectra of the (b, d) Cu 2p and (e) Au 4f electrons in Cu_25_-(Ph)_3_P and AuCu_24_-(Ph)_3_P, respectively. The binding energy was calibrated based on C 1s peak at 284.6 eV.


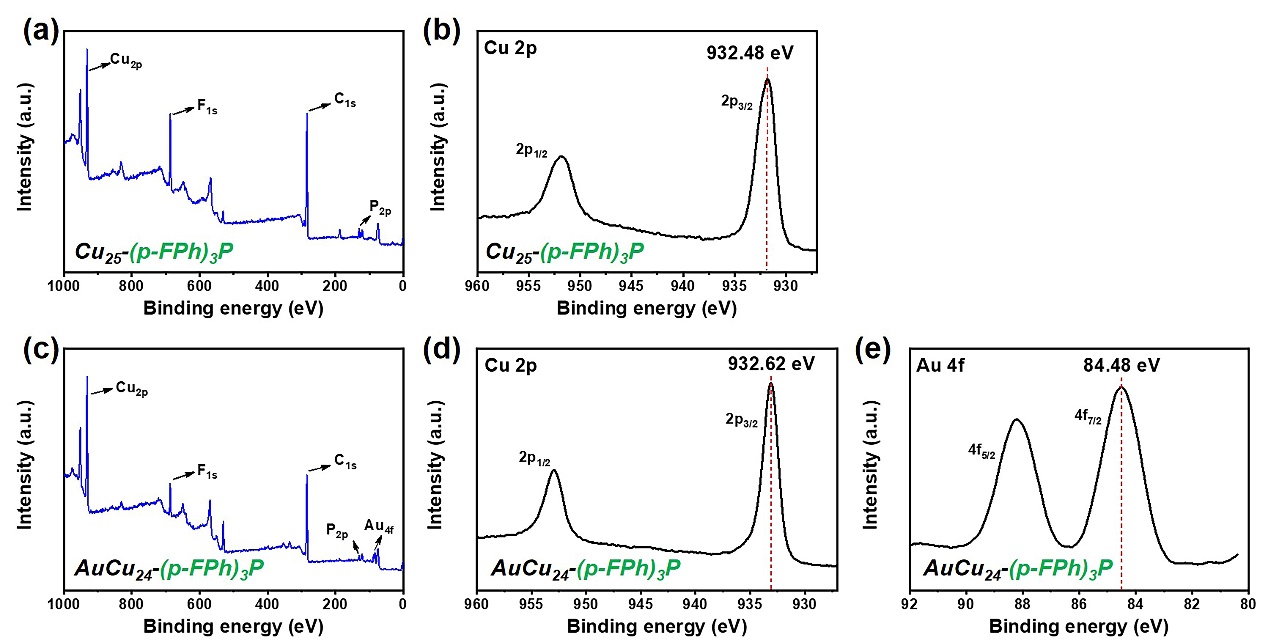


**Supplementary Fig. 4:** (a) The XPS survey scan spectra of (a) Cu_25_-(*p*-FPh)_3_P and (c) AuCu_24_-(*p*-FPh)_3_P. The core-level XPS spectra of the (b, d) Cu 2p and (e) Au 4f electrons in Cu_25_-(*p*-FPh)_3_P and AuCu_24_-(*p*-FPh)_3_P, respectively. The binding energy was calibrated based on C 1s peak at 284.6 eV.

**
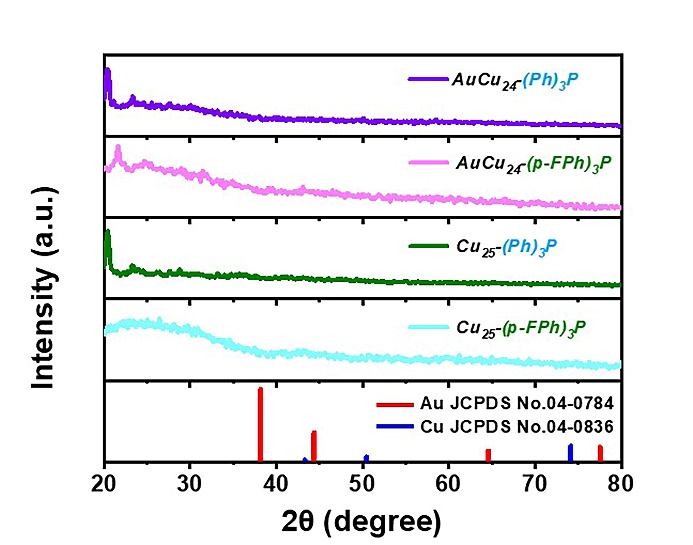
**

**Supplementary Fig. 5:** XRD patterns of the AuCu_24_-(Ph)_3_P/CNTs, Cu_25_-(Ph)_3_P/CNTs, AuCu_24_-(*p*-FPh)_3_P/CNTs and Cu_25_-(*p*-FPh)_3_P/CNTs. No Au or Cu related crystals were observed for the four electrocatalysts.

**
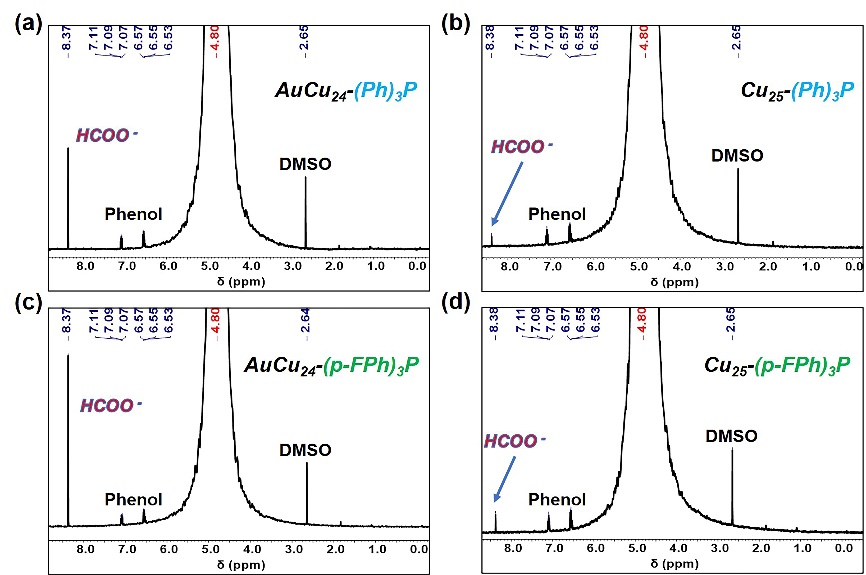
**

**Supplementary Fig. 6:** The ^1^H-NMR spectra of the eCO_2_RR product in liquid phase for (a) AuCu_24_-(Ph)_3_P, (b) Cu_25_-(Ph)_3_P, (c) AuCu_24_-(*p*-FPh)_3_P and (d) Cu_25_-(*p*-FPh)_3_P at -0.8 V (vs. RHE).


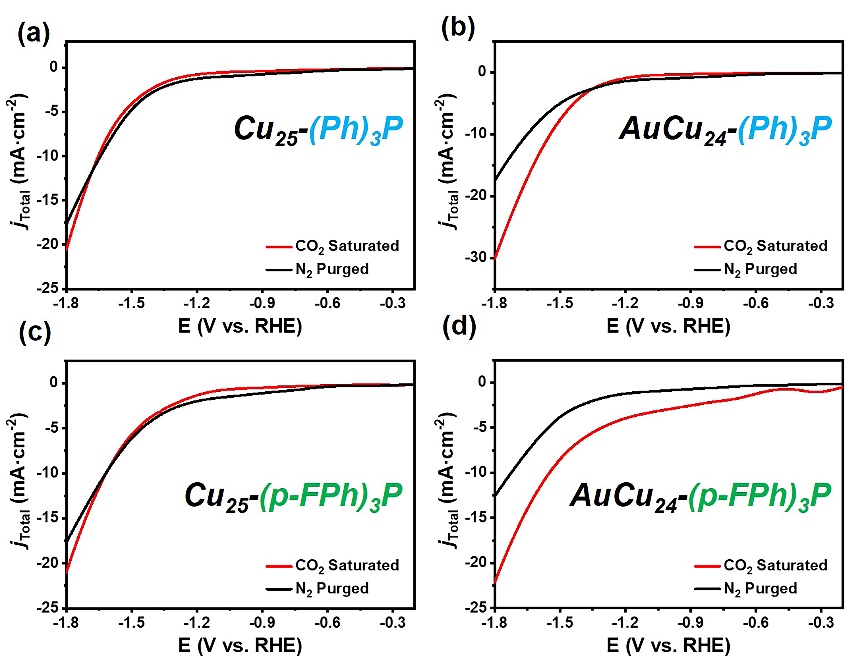


**Supplementary Fig. 7:** The LSV curves of (a) Cu_25_-(Ph)_3_P/CNTs, (b) AuCu_24_-(Ph)_3_P/CNTs, (c) Cu_25_-(*p*-FPh)_3_P/CNTs and (d) AuCu_24_-(*p*-FPh)_3_P/CNTs in an N_2_ (black) and a CO_2_ (red) saturated 0.5 M KHCO_3_ solution.


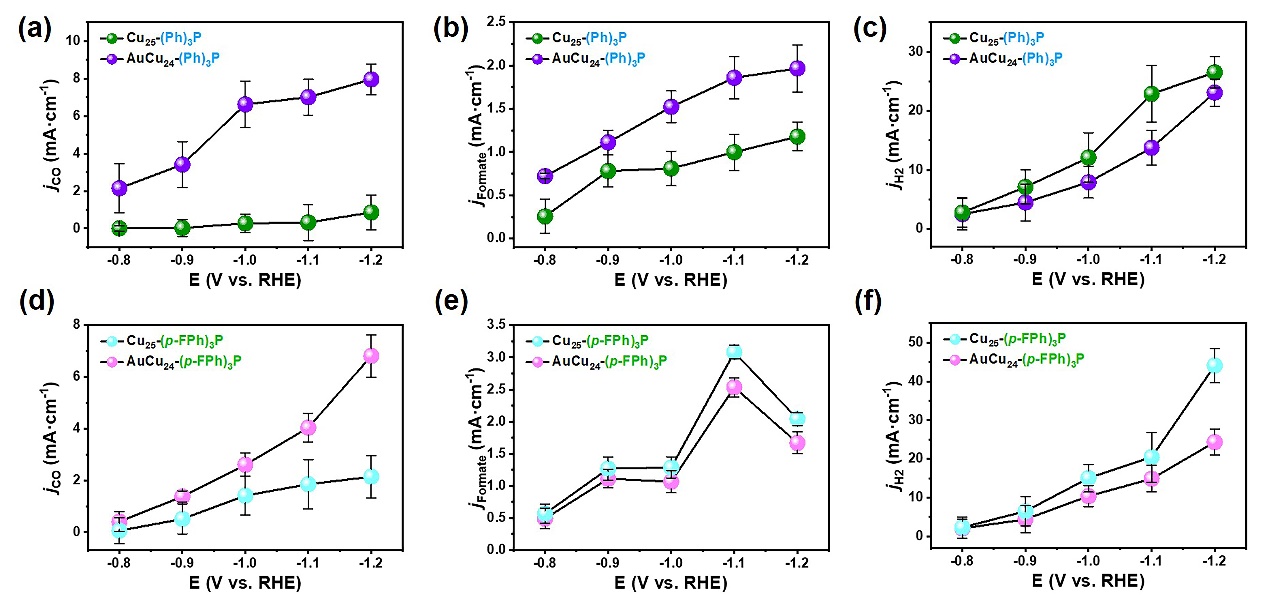


**Supplementary Fig. 8:** (a, d) CO, (b, e) Formate and (c, f) H_2_ partial current density for Cu_25_-(Ph)_3_P/CNTs, AuCu_24_-(Ph)_3_P/CNTs, Cu_25_-(*p*-FPh)_3_P/CNTs and AuCu_24_-(*p*-FPh)_3_P/CNTs examined at different applied potentials. The error bars represent the standard deviation of three tests at the same test potential.


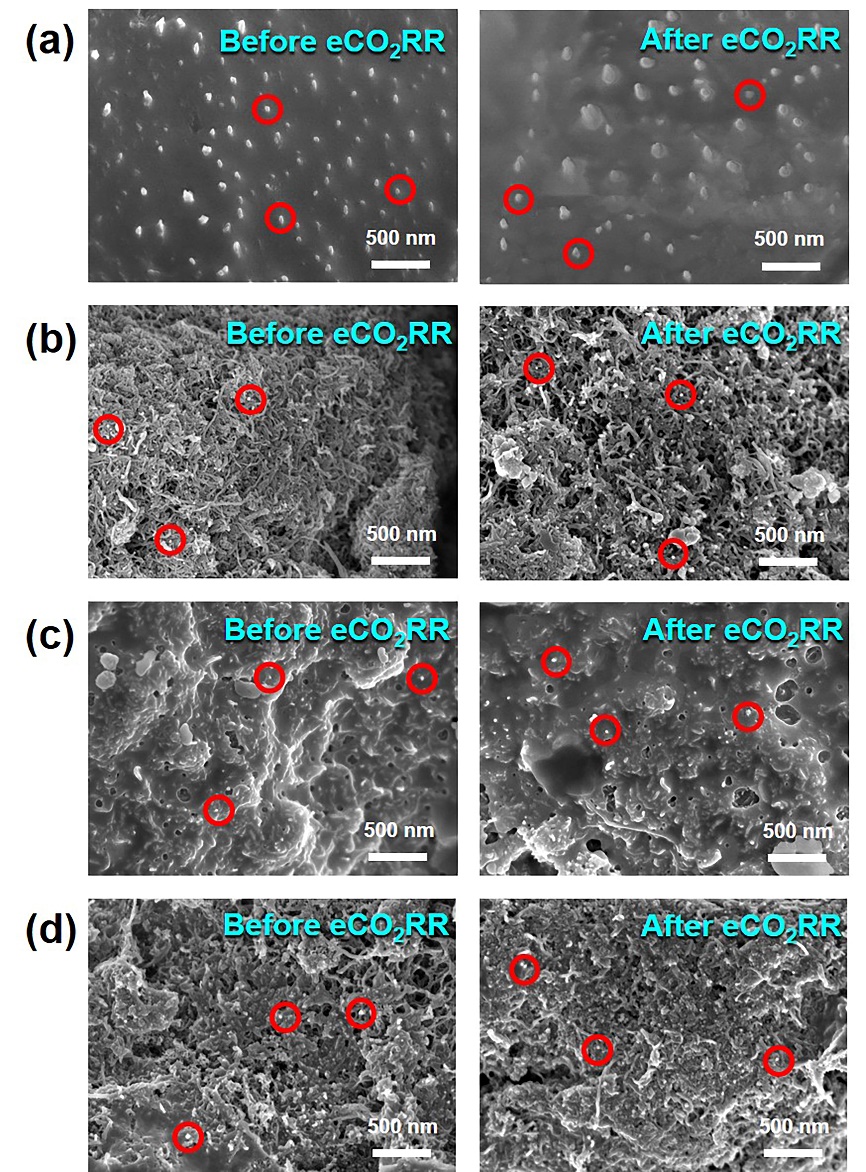


**Supplementary Fig. 9:** The morphology of (a) Cu_25_-(Ph)_3_P/CNTs, (b) AuCu_24_-(Ph)_3_P/CNTs, (c) Cu_25_-(*p*-FPh)_3_P/CNTs and (d) AuCu_24_-(*p*-FPh)_3_P/CNTs before (left) and after (right) eCO2RR at -0.8 V.


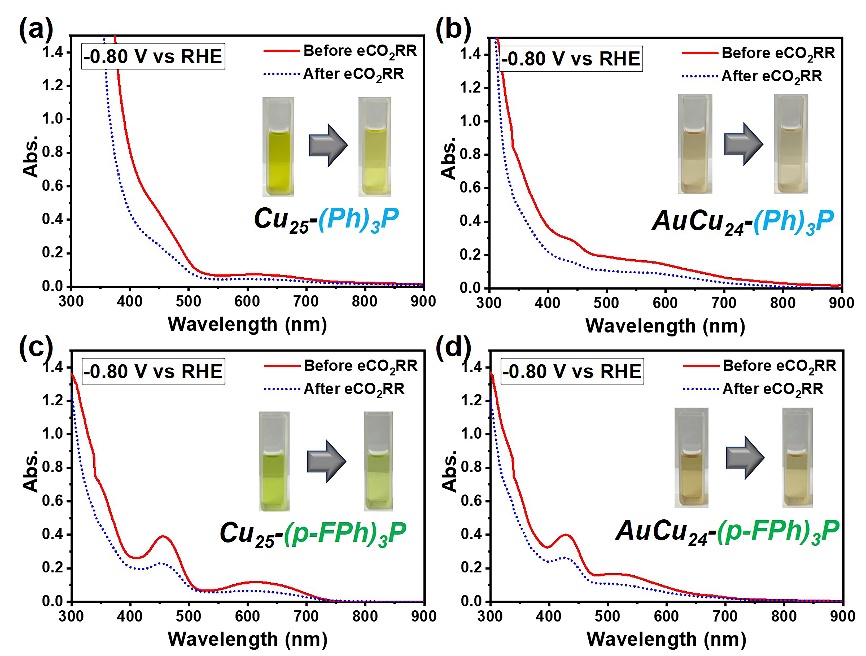


**Supplementary Fig. 10:** UV-vis absorbance spectra of the clusters before and after eCO2RR at -0.8 V for 5h: (a) Cu_25_-(Ph)_3_P/CNTs, (b) AuCu_24_-(Ph)_3_P/CNTs, (c) Cu_25_-(*p*-FPh)_3_P/CNTs and (d) AuCu_24_-(*p*-FPh)_3_P/CNTs.


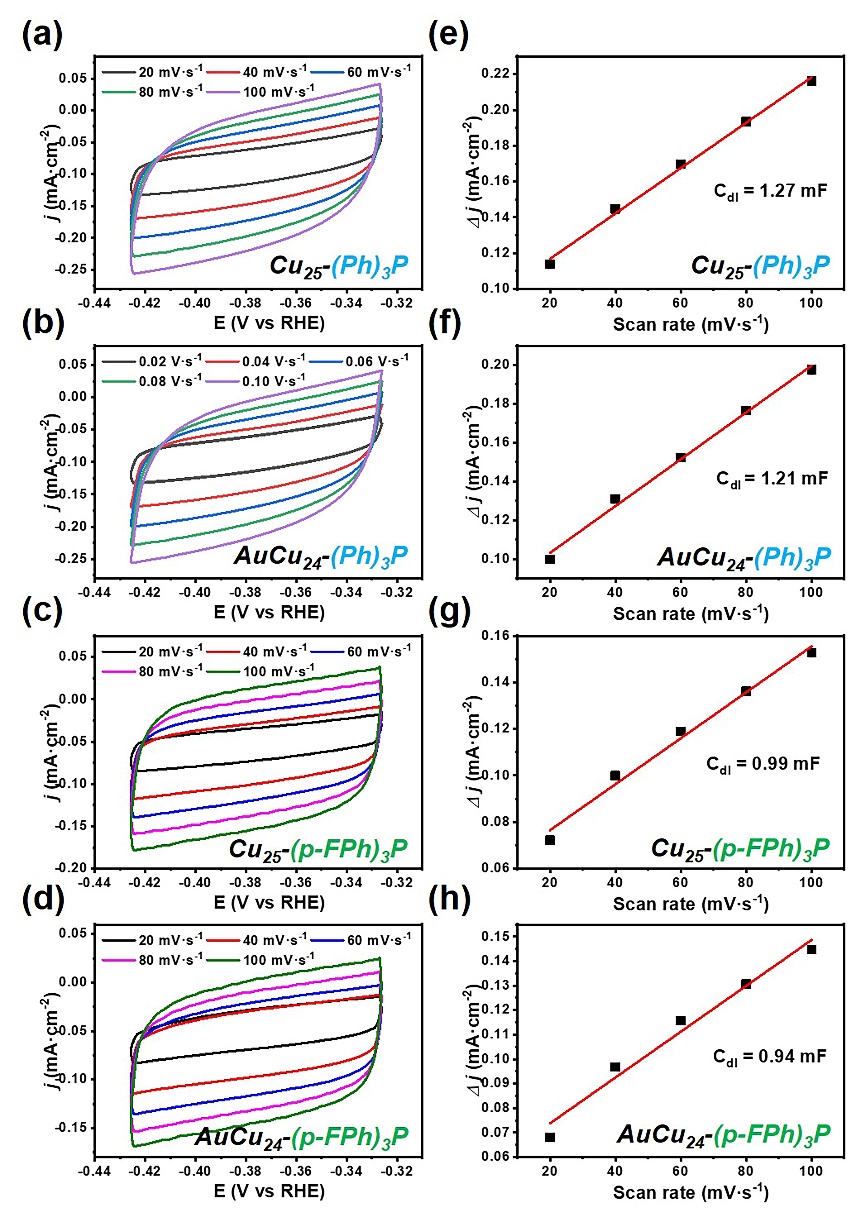


**Supplementary Fig. 11:** Cyclic voltammetry of (a) Cu_25_-(Ph)_3_P/CNTs, (b) AuCu_24_-(Ph)_3_P/CNTs, (c) Cu_25_-(*p*-FPh)_3_P/CNTs and (d) AuCu_24_-(*p*-FPh)_3_P/CNTs in 0.5 M KHCO_3_ electrolyte at a scan rate from 20, 40, 60, 80, 100 mV·s^–1^. The corresponding linear curves of current density and scan rate for (e) Cu_25_-(Ph)_3_P/CNTs, (f) AuCu_24_-(Ph)_3_P/CNTs, (g) Cu_25_-(*p*-FPh)_3_P/CNTs and (h) AuCu_24_-(*p*-FPh)_3_P/CNTs.


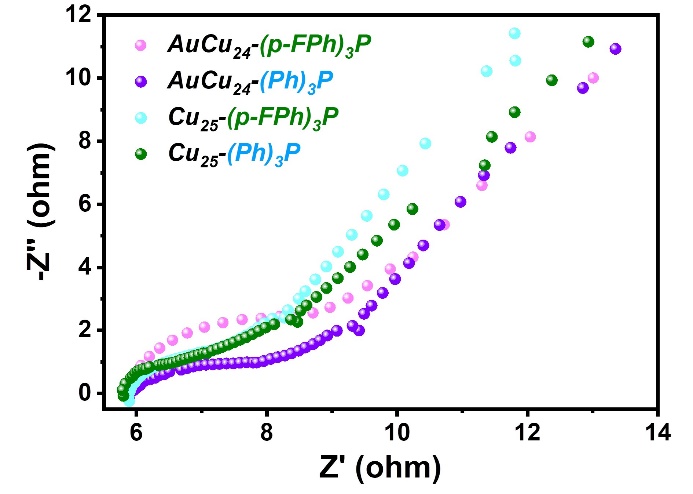


**Supplementary Fig. 12:** Electrochemical impedance spectra (EIS) of Cu_25_-(Ph)_3_P/CNTs, AuCu_24_-(Ph)_3_P/CNTs, Cu_25_-(*p*-FPh)_3_P/CNTs and AuCu_24_-(*p*-FPh)_3_P/CNTs.

**Supplementary Tables**

**Supplementary Table 1:** The XPS data of Cu_25_-(Ph)_3_P, AuCu_24_-(Ph)_3_P, Cu_25_-(*p*-FPh)_3_P and AuCu_24_-(*p*-FPh)_3_P NCs.

| **Sample** | **Au 4f_5/2_**  **(eV)** | **Au 4f_7/2_**  **(eV)** | **Cu 2p_1/2_**  **(eV)** | **Cu 2p_3/2_**  **(eV)** | **Au/Cu ratio** | |
| --- | --- | --- | --- | --- | --- | --- |
|  |  |  |  |  | **Exp.** | **Cal.** |
| Cu_25_-(Ph)_3_P | **/** | **/** | 952.33 | 932.42 | **/** | **/** |
| AuCu_24_-(Ph)_3_P | 87.75 | 84.14 | 952.47 | 932.56 | 1.03/ 24.15 | 1/ 24 |
| Cu_25_-(*p*-FPh)_3_P | / | / | 952.39 | 932.48 | / | / |
| AuCu_24_-(*p*-FPh)_3_P | 88.09 | 84.48 | 952.53 | 932.62 | 1.16/ 24.21 | 1/ 24 |

**Supplementary Table 2:** Recovery of the four electrocatalysts after eCO_2_RR test at -0.80 V.

| **Catalysts** | **Wavelength**  **(*λ*_max_, nm)** | **Absorbance before reaction**  **(A)** | **Absorbance after reaction**  **(A)** | **Recovery**  **(%)** |
| --- | --- | --- | --- | --- |
| Cu_25_-(Ph)_3_P | 635 | 0.08 | 0.04 | 50.1% |
| AuCu_24_-(Ph)_3_P | 580 | 0.16 | 0.09 | 56.3% |
| Cu_25_-(*p*-FPh)_3_P | 454 | 0.38 | 0.23 | 60.5% |
| AuCu_24_-(*p*-FPh)_3_P | 423 | 0.40 | 0.27 | 67.5% |
