# Supplementary material for: Effects of ligand tuning and core doping of atomically precise copper nanoclusters on CO2 electroreduction selectivity
Source: Commun Chem. 2022 Dec 19;5:172. doi: 10.1038/s42004-022-00779-0 (PMC9814636; doi:10.1038/s42004-022-00779-0)
Supplement: Supplementary file 1 — Wang_PR File [file 42004_2022_779_MOESM1_ESM.pdf]

Peer Review File

Effects of ligand tuning and core doping of atomically precise copper nanoclusters on CO2 electroreduction selectivity

Open Access This file is licensed under a Creative Commons Attribution 4.0 International License, which permits use, sharing, adaptation, distribution and reproduction in any medium or format, as long as you give appropriate credit to the original author(s) and the source, provide a link to the Creative Commons license, and indicate if changes were made. In the cases where the authors are anonymous, such as is the case for the reports of anonymous peer reviewers, author attribution should be to 'Anonymous Referee' followed by a clear attribution to the source work. The images or other third party material in this file are included in the article's Creative Commons license, unless indicated otherwise in a credit line to the material. If material is not included in the article's Creative Commons license and your intended use is not permitted by statutory regulation or exceeds the permitted use, you will need to obtain permission directly from the copyright holder. To view a copy of this license, visit http://creativecommons.org/licenses/by/4.0/.

Reviewers' comments:

Reviewer #1 (Remarks to the Author):

The manuscript by Ding et al. reports single-atom doping effect and ligand effect on CO2 electroreduction (CO2RR) by comparing monogold-doped Au1Cu24 and homocopper Cu25 nanoclusters protected by triphenylphosphine or/and tris(4-fluorophenyl)phosphine. Several issues need to be further clarified. The manuscript can only be considered after the authors address the following points.

Q1: Compared with Figure 2, the partial current density in Figure S7 seems to miss the error bar? And the equation S3 makes a mistake that "=" missing?

Q2: What is the different between Qt (the total charge consumed) and C (total charge consumed in electrochemical reaction)? How to calculate them?

Q3: The 5-h long-term stability measurement is too short, please prolong it and add the corresponding FEs.

Q4: In the introduction section, some recently reported metal clusters-based catalysts toward electrochemical reactions are suggested to be cited, such as Small 2022, 2104965; Appl. Catal. B: Environ. 2022, 307, 121145; Small Structures 2022, doi.org/10.1002/sstr.202200086; Nano Res. 2022, doi.org/10.1007/s12274-022-4929-7; Nano Res. 2022, 15, 5842. Of course, some Cu-based catalysts for eCO2RR are also should be cited, such as Nano Research Energy. 2022, 1: e9120015; Nano Research Energy. 2022, 1: e9120021.

Q5: Similar Figure 2, the error bars need to be added in Figures 3b, 3c, 3e and 3e.

Q6: The electrochemical impedance spectroscopy (EIS) and X-ray diffraction (XRD) characterization relating to Cu25-(Ph)3P, Cu25-(pFPh)3P, AuCu24-(Ph)3P and AuCu24-(p-FPh)3P catalysts need to be provided in Supporting Information.

Q7: The current density corrected by ECSA can reflect the intrinsic activity of Cu25-(Ph)3P, Cu25-(pFPh)3P, AuCu24-(Ph)3P and AuCu24-(p-FPh)3P catalysts, please give current density after ECSA correction and reasonable analysis.

Reviewer #2 (Remarks to the Author):

Recommendation: May be suitable for Communications Chemistry after small revisions.
Comments:
In this work, the authors presented a case for the impact of electron configuration modulating over atomically precise copper nanocluster on electroreduction selectivity. They also found that the four clusters exhibited different catalytic performances toward CO2RR, in which single-atom doping clusters converts CO2 into CO and formate, while hydrogen evolution reaction are the main reduction prossess for homocopper Cu25 NCs. And the inductive effect of ligand strongly favors the formation of formate in CO2RR. Overall, it is a high quality work. This manuscript is recommended for publication if the following concerns can be addressed:
1) Please describe the FE calculation method in detail, including the collected method for gas products.
2) The four clusters were respectively deposited onto acid multi-walled carbon nanotubes support, I want to understand whether there is an interaction between nanoclusters and support. Why did the author use carbon nanotubes rather than carbon black?
3) Furthermore, the stability of the nanoclusters before and after electrocatalysis was characterized by UV-vis spectra (Figure S9). I want to know what causes the subtle differences of absorbance profile of

nanoclusters before and after electrocatalysis?
4) There are some small mistakes in the current manuscripts as follow:
a. The description of Figure S7 is incorrect.
b. It is better to make the abbreviation of CO2 electroreduction (eCO2RR) unified.
c. The author should point out the test potential of the 1H-NMR spectra of the eCO2RR product in liquid phase for the four clusters.

Reviewer #3 (Remarks to the Author):

Recommendation: Minor revision
Comments:
Mei Ding and coworkers present that the singe-atom doping effect and inductive effect on CO2 reduction by comparing with four M@Cu24 nanoclusters (protected by triphenylphosphine or tris(4-fluorophenyl)phosphine) that share a similar M@Cu12 core structure, and the results demonstrate that single-atom substitution can substantially improve CO2 electroreduction activity and selectivity. The authors did a good job on explaining the production method and investigating the impact of electron configuration modulating on CO2 reduction. The story is well-organized. My recommendation is minor revision. There are some minor issues must be addressed prior to publish as an article in Communications Chemistry:
1. The stability is one of the important indexes to evaluate the catalyst material. The authors provide the stability test of four clusters at -0.8 V vs. RHE for 5 h. I want to know why did the author chose the stability test of four clusters at -0.8 V?
2. The resolution of figures in supporting information is too low to be seen clearly. It is better to improve the definition of the figures to clearly express subtle differences.
3. There are some small mistakes and typos. E. g. expression of sentence, such as "Figure S2b" should be "Figure S2c"; the "NC" should be "NCs"; there is lack of "=" in equation of S3; journal names should be correctly in reference; the "Cupric (II) acetylacetonate" should be "cupric (II) acetylacetonate"; "Synthesis of [Cu25H22(Ph3P)12]+ nanocluster" should be "Synthesis of [Cu25H22(Ph3P)12]+ nanoclusters" The authors must check the whole manuscript carefully to make sure to avoid these errors.
In summary, this manuscript currently has basically satisfied the high scientific standard and high impact demand of Communications Chemistry, hence a minor revision is recommended at this stage. After the above issue have been unambiguously addressed, it can be considered to publish in Communications Chemistry.

We thank the reviewers for the constructive comments. We have addressed all the comments point-to-point unambiguously and made corresponding changes in the text. The relevant texts are highlighted in red in the revised manuscript and supporting information. The comments are in blue and the responses are in black listed as follows.

Reviewer #1:

The manuscript by Ding et al. reports single-atom doping effect and ligand effect on CO_2 electroreduction (CO_2RR) by comparing monogold-doped Au_1Cu_{24} and homocopper Cu_{25} nanoclusters protected by triphenylphosphine or/and tris(4-fluorophenyl)phosphine. Several issues need to be further clarified. The manuscript can only be considered after the authors address the following points.

Q1: Compared with Figure 2, the partial current density in Figure S7 seems to miss the error bar? And the equation S3 makes a mistake that "=" missing?

Response: Thanks a lot. We have replaced original Figure S7 with partial current density with error bar in new Figure S7. And we have corrected the mistakes in equation S3. Furthermore, we have carefully checked and corrected all mistakes like this in the whole manuscript.

Q2: What is the different between Qt (the total charge consumed) and C (total charge consumed in electrochemical reaction)? How to calculate them?

Response: We are truly sorry for that. Both Qt and C are defined as the total amount of electricity consumed in electrochemical reactions, which were recorded by electrochemical workstation. Therefore, we have removed "C" to ensure uniform expression. Furthermore, we have carefully checked and corrected all the small mistakes in the whole manuscript.

Q3: The 5-h long-term stability measurement is too short, please prolong it and add the corresponding FEs.

Response: Thanks for the kind comment. We have prolonged the time of stability measurement to 12 h. And we have re-drawn the Figure 3 and added corresponding descriptions in the revised manuscript.

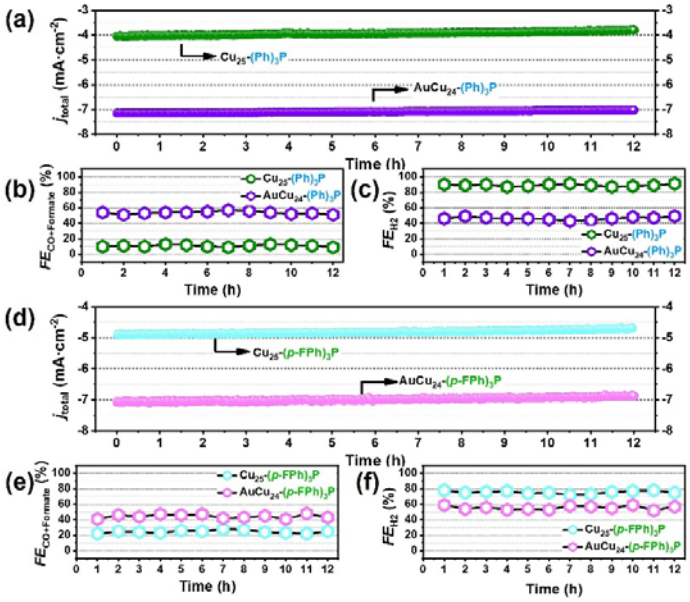

Figure 3. Long-term stability of *Cu_{25}-(Ph)$_3$P*, *$AuCu_{24}$-(Ph)$_3$P*, *Cu_{25}-(p-FPh)$_3$P* and *$AuCu_{24}$-(p-FPh)$_3$P* at -0.80 V. (a, d) *i*-t curve; *FE*s of (b, e) C1 products and (c, f) H_2 at different time.

Concretely, as illustrated in **Figure 3a** and **3d**, the current density and corresponding *FE* value of two mono-Au doped NCs remained almost unchanged (j_{total}= -7.13 to -6.91 mA·cm^{-2} for *$AuCu_{24}$-(Ph)$_3$P/CNTs*; j_{total} from -7.05 to -6.90 mA·cm^{-2} for *$AuCu_{24}$-(p-FPh)$_3$P/CNTs*) after 12 hour of continuous operation, indicating robust long-term durability. However, under the same conditions, the current density of *Cu_{25}-(Ph)$_3$P/CNTs* and *Cu_{25}-(p-FPh)$_3$P/CNTs* decreased about 5.7% (from -4.04 to -3.81 mA·cm^{-2}) and 4.3% (from -4.90 to -4.69 mA·cm^{-2}), respectively.

Q4: In the introduction section, some recently reported metal clusters-based catalysts toward electrochemical reactions are suggested to be cited, such as Small 2022, 2104965; Appl. Catal. B: Environ. 2022, 307, 121145; Small Structures 2022, doi.org/10.1002/sstr.202200086; Nano Res. 2022, doi.org/10.1007/s12274-022-4929-7; Nano Res. 2022, 15, 5842. Of course, some Cu-based catalysts for eCO2RR are also should be cited, such as Nano Research Energy. 2022, 1: e9120015; Nano Research Energy. 2022, 1: e9120021.

Response: Thanks for the suggestion. These papers are indeed important and closely relevant, and they have been cited in the newly revised manuscript (*Appl. Catal. B: Environ.* **2022**, *307*, 121145, Ref. 9; *Nano Res.* **2022**, *15*, 5842, Ref. 12; *Nano Res. Energy*. **2022**, *1*: e9120015, Ref. 33; *Nano Res. Energy* **2022**, *1*: e9120021, Ref. 34). Meanwhile, we have rewritten this sentence and marked in red. Please see the modified one.

Q5: Similar Figure 2, the error bars need to be added in Figures 3b, 3c, 3e and 3e.

Response: Thanks for the kind comments. The long-term stability test of *Cu_{25}-(Ph)$_3$P*, *$AuCu_{24}$-(Ph)$_3$P*, *Cu_{25}-(p-FPh)$_3$P* and *$AuCu_{24}$-(p-FPh)$_3$P* at -0.80 V for 12h. However, in the Figure 3, the H_2, CO and formate faradaic efficiency (FE) of the four NCs were recorded at one-hour intervals by gas chromatography, unlike the three parallel FEs at different test potential in the Figure 2.

Q6: The electrochemical impedance spectroscopy (EIS) and X-ray diffraction (XRD) characterization relating to Cu_{25}-(Ph)$_3$P, Cu_{25}-(*p*-FPh)$_3$P, $AuCu_{24}$-(Ph)$_3$P and $AuCu_{24}$-(*p*-FPh)$_3$P catalysts need to be provided in Supporting Information.

Response: Thanks for the suggestion. we have conducted the electrochemical impedance spectroscopy and X-ray diffraction (XRD) characterization of Cu_{25}-(Ph)$_3$P, Cu_{25}-(*p*-FPh)$_3$P, $AuCu_{24}$-(Ph)$_3$P and $AuCu_{24}$-(*p*-FPh)$_3$P, and have added the XRD (Figure S5) and EIS (Figure S12) and in supporting information.

Concretely, no nanoparticles were observed on the surface of four electrocatalysts, which corresponds to the X-ray diffraction (XRD) pattern (**Figure S5**). Furthermore, the electrochemical impedance (EIS) was carried out to explore the charge transport properties of the four NCs at the electrode/electrolyte interface (**Figure S12**). The Nyquist plot of both homogeneous Cu_{25} NCs exhibited a much smaller the semicircular diameters than that of mono-gold-doped $AuCu_{24}$, and the former has a conductivity with lower interfacial charge–transfer resistance.

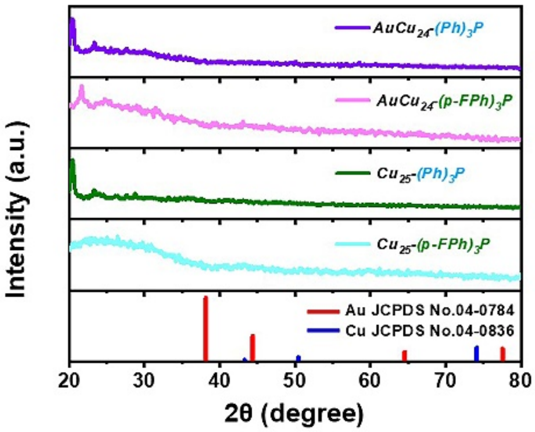

Figure S5. XRD patterns of the *$AuCu_{24}$-(Ph)$_3$P/CNTs*, *Cu_{25}-(Ph)$_3$P/CNTs*, *$AuCu_{24}$-(p-FPh)$_3$P/CNTs* and *Cu_{25}-(p-FPh)$_3$P/CNTs*. No Au or Cu related crystals were observed for the four electrocatalysts.

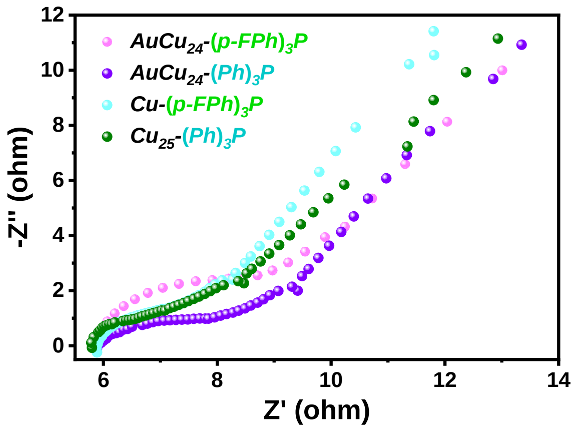

Figure S12. Electrochemical impedance spectra (EIS) of *Cu_{25}-(Ph)$_3$P*, *$AuCu_{24}$-(Ph)$_3$P*, *Cu_{25}-(p-FPh)$_3$P* and *$AuCu_{24}$-(p-FPh)$_3$P*.

Q7: The current density corrected by ECSA can reflect the intrinsic activity of Cu_{25}-(Ph)$_3$P, Cu_{25}-(*p*-FPh)$_3$P, $AuCu_{24}$-(Ph)$_3$P and $AuCu_{24}$-(*p*-FPh)$_3$P catalysts, please give current density after ECSA correction and reasonable analysis.

Response: Thanks for the kind suggestion. we have added the ECSA of Cu_{25}-(Ph)$_3$P, Cu_{25}-(*p*-FPh)$_3$P, $AuCu_{24}$-(Ph)$_3$P and $AuCu_{24}$-(*p*-FPh)$_3$P catalysts in supporting information (**Figure S10**). And the corresponding descriptions were added and marked in red in manuscript.

Concretely, the electrochemically active surface area (ECSA) was measured to further reveal the reason for the difference in catalytic performance of the four clusters in eCO_2RR. The current density of *Cu_{25}-(Ph)$_3$P*, *$AuCu_{24}$-(Ph)$_3$P*, *Cu_{25}-(p-FPh)$_3$P* and *$AuCu_{24}$-(p-FPh)$_3$P* at different scan rate ranging from 20 to 100 mV·s^{-1} were recorded and shown in Figures S10, and based on that, the double-layer capacitance (C_{dl}) of the four NCs were evaluated to be 1.27, 1.21, 0.99 and 0.94 mF, respectively. Thus, the ECSA of *Cu_{25}-(Ph)$_3$P*, *$AuCu_{24}$-(Ph)$_3$P*, *Cu_{25}-(p-FPh)$_3$P* and *$AuCu_{24}$-(p-FPh)$_3$P* were calculated to be 31.75, 30.25, 24.75 and 23.50 cm^2, respectively. Therefore, the number of active sites of the three clusters can be arranged in descending order as *Cu_{25}-(Ph)$_3$P* > *$AuCu_{24}$-(Ph)$_3$P* > *Cu_{25}-(p-FPh)$_3$P* > *$AuCu_{24}$-(p-FPh)$_3$P*, which was attributed to the number of active sites scales with the ligand removal.

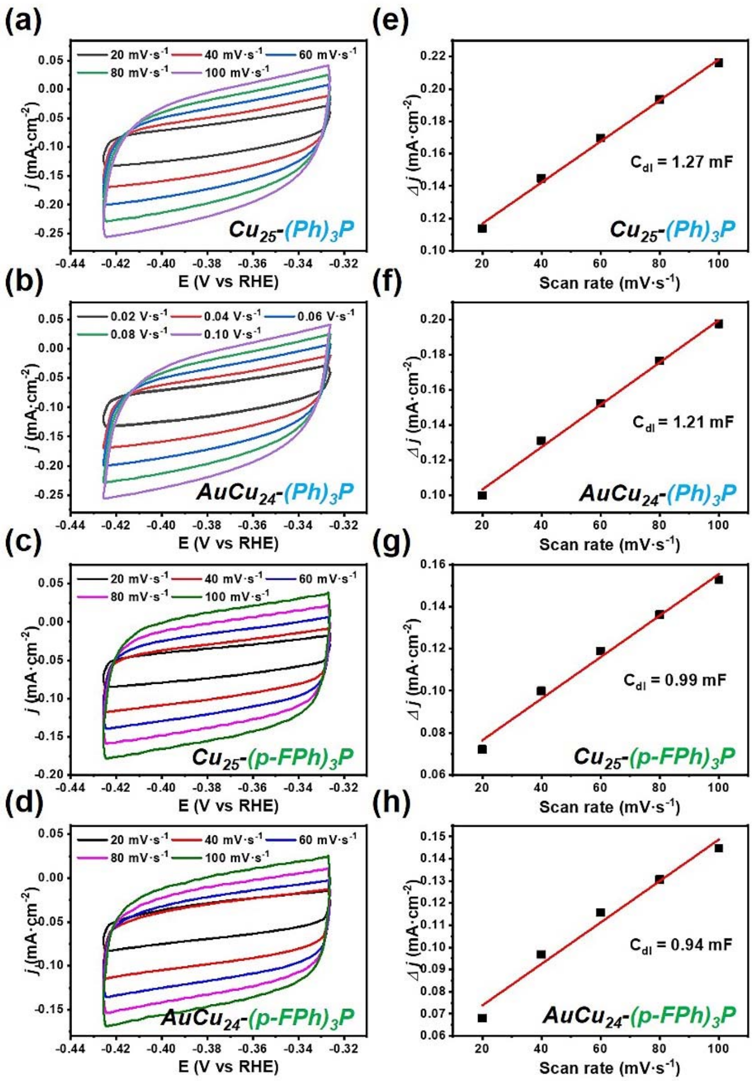

Figure S11. Cyclic voltammetry of (a) *Cu25-(Ph)3P/CNTs*, (b) *AuCu24-(Ph)3P/CNTs*, (c) *Cu25-(p-FPh)3P/CNTs* and (d) *AuCu24-(p-FPh)3P/CNTs* in 0.5 M $KHCO_3$ electrolyte at a scan rate from 20, 40, 60, 80, 100 mV s^{-1}. The corresponding linear curves of current density and scan rate for (e) *Cu25-(Ph)3P/CNTs*, (f) *AuCu24-(Ph)3P/CNTs*, (g) *Cu25-(p-FPh)3P/CNTs* and (h) *AuCu24-(p-FPh)3P/CNTs*.

Reviewer #2

Recommendation: May be suitable for Communications Chemistry after small revisions.

Comments:

In this work, the authors presented a case for the impact of electron configuration modulating over atomically precise copper nanocluster on electroreduction selectivity. They also found that the four clusters exhibited different catalytic performances toward CO_2RR, in which single-atom doping clusters converts CO_2 into CO and formate, while hydrogen evolution reaction are the main reduction prossess for homocopper Cu_{25} NCs. And the inductive effect of ligand strongly favors the formation of formate in CO_2RR. Overall, it is a high quality work. This manuscript is recommended for publication if the following concerns can be addressed:

1) Please describe the FE calculation method in detail, including the collected method for gas products.

Response: Thanks for the comment. We have added the details of FE calculation in the Electrochemical measurements and marked in red. The output of the gas flow from the cathode chamber didn't be collected by us, while it was in situ online directed into a gas chromatograph instrument (GC3900Plus, RUI NENG) for identification and quantification of the gaseous products, which was purged for 30 min with an average rate of 10 $mL{\cdot}min^{-1}$ (at room temperature and ambient pressure) prior to the test.

2) The four clusters were respectively deposited onto acid multi-walled carbon nanotubes support, I want to understand whether there is an interaction between nanoclusters and support. Why did the author use carbon nanotubes rather than carbon black?

Response: Thanks a lot. Due to the small size of the clusters (size <3 nm), they can be stably bound to carbon nanotubes by physical adsorption. Compared with carbon black, the surface of acid multi-walled carbon nanotubes possesses much more defects, which can contribute to CO_2 enrichment. Meanwhile, the holes between carbon fibers can increase CO_2 penetration, which in turn improves the contact between the catalyst and CO_2. Therefore, we have chosen acid multi-walled carbon nanotubes as conductive support rather than carbon black.

3) Furthermore, the stability of the nanoclusters before and after electrocatalysis was characterized by UV-vis spectra (Figure S9). I want to know what causes the subtle differences of absorbance profile of nanoclusters before and after electrocatalysis?

Response: Thanks for the kind comments. After CO_2RR, the partial clusters on carbon cloth were dissolved in dichloromethane directly, leading to the absorbance of clusters were slightly decreased. Furthermore, decomposition was occurred in partial clusters in the catalytic process, resulting in subtle changes of absorbance before and after CO_2RR.

4) There are some small mistakes in the current manuscripts as follow:

a. The description of Figure S7 is incorrect.

Response: Thanks a lot. We have carefully checked and corrected all small mistakes in the whole manuscript.

b. It is better to make the abbreviation of CO_2 electroreduction (eCO_2RR) unified.

Response: Thanks. We have carefully checked and corrected all abbreviation such as "eCO_2RR" in the whole manuscript to ensure them unified.

c. The author should point out the test potential of the ^{1}H-NMR spectra of the eCO_2RR product in liquid phase for the four clusters.

Response: Thanks for the kind suggestion. We have added the test potential (-0.8 V (vs. RHE)) of the ^{1}H-NMR spectra of the eCO_2RR product in liquid phase for the four clusters.

Reviewer #3

Recommendation: Minor revision

Comments:

Mei Ding and coworkers present that the singe-atom doping effect and inductive effect on CO_2 reduction by comparing with four M@Cu_{24} nanoclusters (protected by triphenylphosphine or tris(4-fluorophenyl)phosphine) that share a similar M@Cu_{12} core structure, and the results demonstrate that single-atom substitution can substantially improve CO_2 electroreduction activity and selectivity. The authors did a good job on explaining the production method and investigating the impact of electron configuration modulating on CO_2 reduction. The story is well-organized. My recommendation is minor revision. There are some minor issues must be addressed prior to publish as an article in Communications Chemistry:

1. The stability is one of the important indexes to evaluate the catalyst material. The authors provide the stability test of four clusters at -0.8 V vs. RHE for 5 h. I want to know why did the author chose the stability test of four clusters at -0.8 V?

Response: Thanks for the comment. By comparing the FEs of each product of the eCO_2RR at different potentials, we found that the catalytic performance of these catalysts can be distinguished at -0.8 V vs. RHE. And the catalytic performance of eCO_2RR of each catalyst at -0.8 V vs. RHE is better than other potentials, so the stability test is chosen at -0.8 V vs. RHE.

2. The resolution of figures in supporting information is too low to be seen clearly. It is better to improve the definition of the figures to clearly express subtle differences.

Response: Thanks for the kind comment. We have re-drawn all figures in the supporting information to improve the definition of the figures to clearly express subtle differences.

3. There are some small mistakes and typos. E. g. expression of sentence, such as "Figure S2b" should be "Figure S2c"; the "NC" should be "NCs"; there is lack of "=" in equation of S3; journal names should be correctly in reference; the "Cupric (II) acetylacetonate" should be "cupric (II) acetylacetonate"; "Synthesis of $[Cu_{25}H_{22}(Ph_3P)_{12}]^+$ nanocluster" should be "Synthesis of $[Cu_{25}H_{22}(Ph_3P)_{12}]^+$ nanoclusters" The authors must check the whole manuscript carefully to make sure to avoid these errors.

Response: Thanks for the comment. We have carefully checked the mistakes and marked the corrected in red. Please see the modified one.

In summary, this manuscript currently has basically satisfied the high scientific standard and high impact demand of Communications Chemistry, hence a minor revision is recommended at this stage. After the above issue have been unambiguously addressed, it can be considered to publish in Communications Chemistry.

REVIEWERS' COMMENTS:

Reviewer #1 (Remarks to the Author):

I read the revised manuscript and the authors’ responses to the reviewers' comments carefully. I think the authors have addressed all the reviewers’ concerns very well.

Reviewer #2 (Remarks to the Author):

After careful evaluation of revised manuscript, I believe that the manuscript can be suitable for publication in journal “Communications Chemistry” in presented form. All necessary additions and clarifications are made in the revised manuscript.

Reviewer #3 (Remarks to the Author):

The authors have clearly addressed all the comments and questions from the reviewers, hence I suggest to accept this revised manuscript to be published in Communications Chemistry in the current format.
